# Targeting Neoepitopes to Treat Solid Malignancies: Immunosurgery

**DOI:** 10.3389/fimmu.2021.592031

**Published:** 2021-07-15

**Authors:** Eric de Sousa, Joana R. Lérias, Antonio Beltran, Georgia Paraschoudi, Carolina Condeço, Jéssica Kamiki, Patrícia Alexandra António, Nuno Figueiredo, Carlos Carvalho, Mireia Castillo-Martin, Zhe Wang, Dário Ligeiro, Martin Rao, Markus Maeurer

**Affiliations:** ^1^ ImmunoSurgery Unit, Champalimaud Centre for the Unknown, Lisbon, Portugal; ^2^ Department of Pathology, Champalimaud Clinical Centre, Lisbon, Portugal; ^3^ Digestive Unit, Champalimaud Clinical Centre, Lisbon, Portugal; ^4^ Jiangsu Industrial Technology Research Institute (JITRI), Applied Adaptome Immunology Institute, Nanjing, China; ^5^ Lisbon Centre for Blood and Transplantation, Instituto Português do Sangue e Transplantação (IPST), Lisbon, Portugal; ^6^ I Medical Clinic, Johannes Gutenberg University of Mainz, Mainz, Germany

**Keywords:** T-cells, antigens, TIL, neoepitopes, precision medicine, vaccination, T-cell receptor, immunotherapy

## Abstract

Successful outcome of immune checkpoint blockade in patients with solid cancers is in part associated with a high tumor mutational burden (TMB) and the recognition of private neoantigens by T-cells. The quality and quantity of target recognition is determined by the repertoire of ‘neoepitope’-specific T-cell receptors (TCRs) in tumor-infiltrating lymphocytes (TIL), or peripheral T-cells. Interferon gamma (IFN-γ), produced by T-cells and other immune cells, is essential for controlling proliferation of transformed cells, induction of apoptosis and enhancing human leukocyte antigen (HLA) expression, thereby increasing immunogenicity of cancer cells. TCR αβ-dependent therapies should account for tumor heterogeneity and availability of the TCR repertoire capable of reacting to neoepitopes and functional HLA pathways. Immunogenic epitopes in the tumor-stroma may also be targeted to achieve tumor-containment by changing the immune-contexture in the tumor microenvironment (TME). Non protein-coding regions of the tumor-cell genome may also contain many aberrantly expressed, non-mutated tumor-associated antigens (TAAs) capable of eliciting productive anti-tumor immune responses. Whole-exome sequencing (WES) and/or RNA sequencing (RNA-Seq) of cancer tissue, combined with several layers of bioinformatic analysis is commonly used to predict possible neoepitopes present in clinical samples. At the ImmunoSurgery Unit of the Champalimaud Centre for the Unknown (CCU), a pipeline combining several tools is used for predicting private mutations from WES and RNA-Seq data followed by the construction of synthetic peptides tailored for immunological response assessment reflecting the patient’s tumor mutations, guided by MHC typing. Subsequent immunoassays allow the detection of differential IFN-γ production patterns associated with (intra-tumoral) spatiotemporal differences in TIL or peripheral T-cells versus TIL. These bioinformatics tools, in addition to histopathological assessment, immunological readouts from functional bioassays and deep T-cell ‘adaptome’ analyses, are expected to advance discovery and development of next-generation personalized precision medicine strategies to improve clinical outcomes in cancer in the context of i) anti-tumor vaccination strategies, ii) gauging mutation-reactive T-cell responses in biological therapies and iii) expansion of tumor-reactive T-cells for the cellular treatment of patients with cancer.

## Introduction

‘*Personalized immunotherapy is all the rage, but neoantigen discovery and validation remains a daunting problem’* echoed an Editorial in Nature Biotechnology 2017 (1). Advances in the last three years in whole exome sequencing (WES), RNA sequencing (RNA-Seq) and combinational peptide vaccination trials combined with checkpoint inhibitors addressed some of the unanswered questions and challenges in therapeutic vaccinations using neoepitopes. Biologically and clinically relevant immune responses happen in distinct immunological contexts, they are dependent on antigen processing, presentation and on the available T-cell receptor (TCR) repertoire that is shaped by previous encounters with antigens. The immune synapse between the major histocompatibility complex (MHC)-peptide and TCR interaction is the center of T-cell activation, which is orchestrated by cells of the innate and adaptive immune response that guides and edit neoepitope-specific T-cell responses. We will therefore review various immune cell types that contribute to successful cellular immune responses and expansion of neoepitope-directed T-cells. Finally, we address in practical terms how neoepitopes are identified in cancer tissue specimens starting with immunohistology, WES, RNA-Seq and epitope prediction algorithms using standard prediction programs.

**Figure 1:**
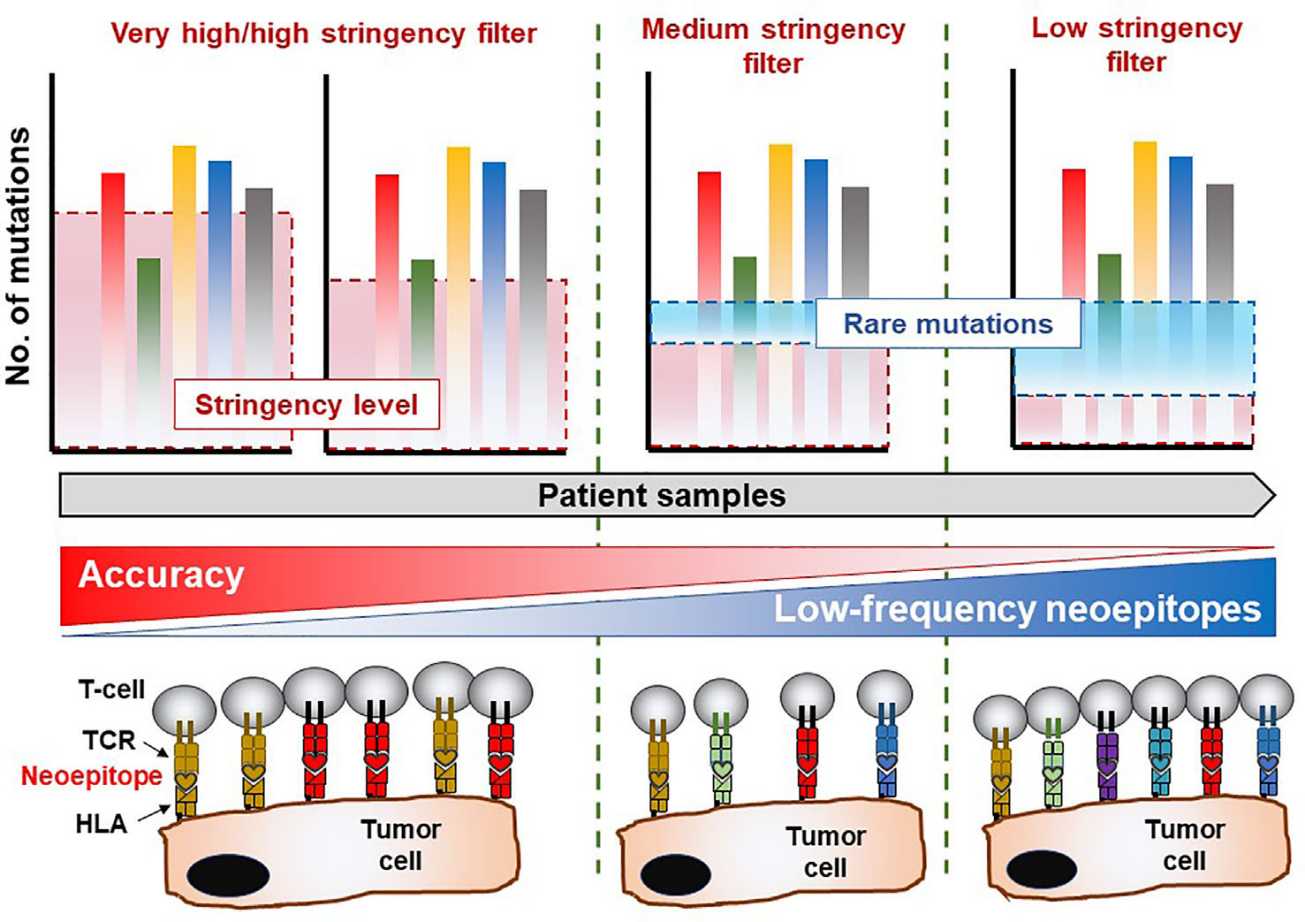
Mutation analysis reveals immune-recognition profile in the TME. Whole-exome sequencing data allows for mining of private somatic mutations in tumor samples compared to healthy (non-transformed) tissue or cells, which is unique to each patient. The stringency of the filtering parameters applied in bioinformatics and statistical analysis of the sequencing data will greatly influence the number of mutations recovered, which are essential for downstream characterization of immune responses of T-cell products. Highly stringent parameters may yield a lower number of mutations albeit with an exceptional level of accuracy. Nevertheless, this approach suffers the risk of overlooking several infrequent mutations which also give rise to immunogenic (T-cell reactive) neoepitopes in the patient. On the contrary, reducing the stringency levels of analysis may reveal rare mutations which facilitate the identification of potentially immunogenic molecular targets recognized by certain TCRs capable of eliciting a biologically relevant anti-tumor immune response. The drawback in the latter scenario is that a high degree of false positive hits may be obtained and included in the final list of legitimate cancer-associated somatic mutations. Thus, a balanced yet wholistic approach is required to identify all immunogenic mutations in tumor tissue which will be instrumental in developing personalized cancer therapies.

**Figure 2:**
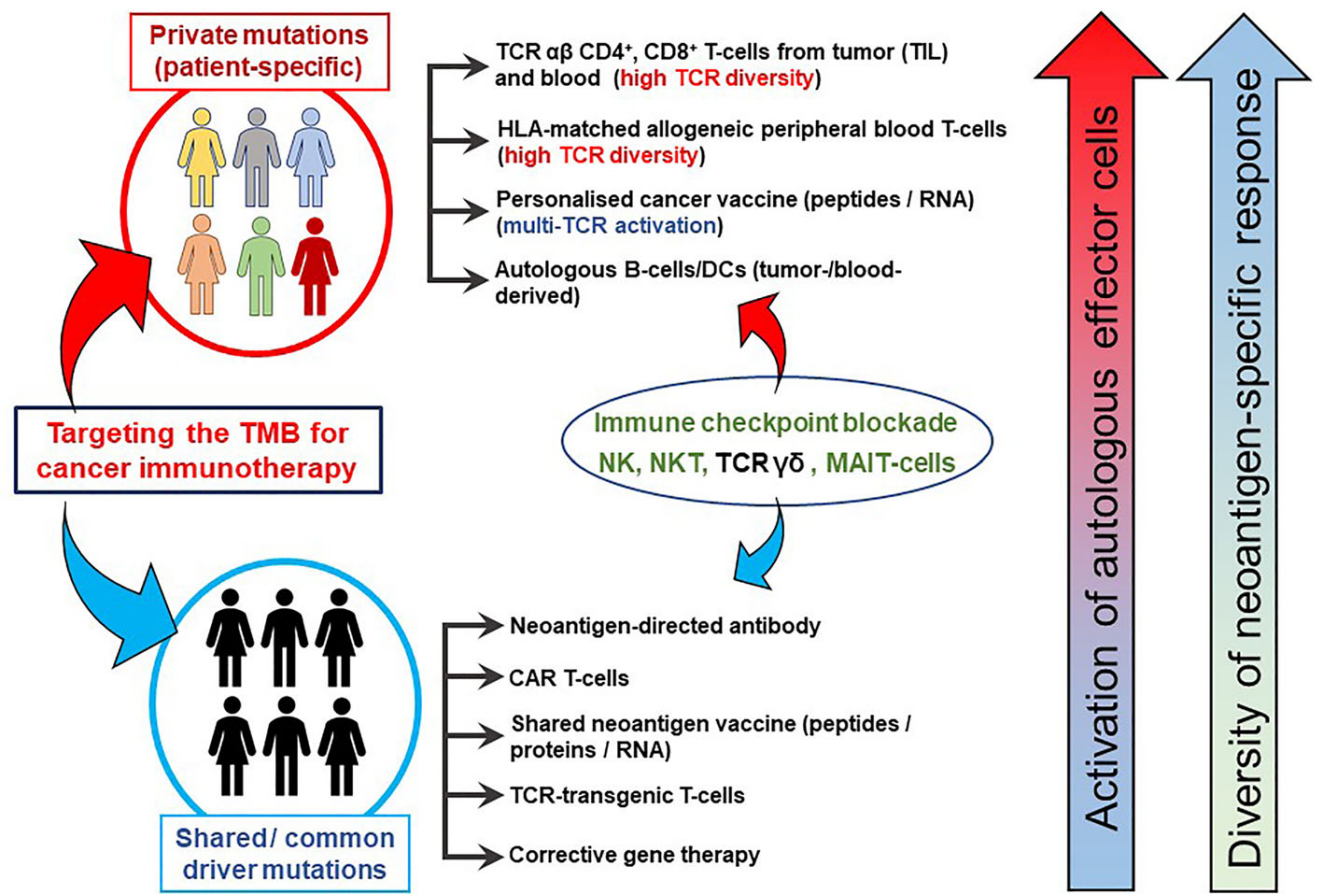
TMB-directed immunotherapy approaches at the Champalimaud Centre for the Unknown. The schematic shows strategies aimed at therapeutic targeting of private (personalized and patient-specific) and shared (often driver) mutations. For personalized therapy, CD4^+^ and CD8^+^ T-cells from TIL or peripheral blood expressing a highly diverse TCR αβ repertoire recognizing a private neoepitopes can be procured. HLA-matched, healthy donor-derived TCRs have also been shown to recognize patient-specific neoepitopes (21). Personalized cancer vaccines, comprising private neoepitopes as a peptide formulation or as RNA constructs, promote durable immune responses in patients with advanced cancer. Autologous B-cells can be used as a source of APCs as well as cytokine producers, in addition to their differentiation into plasma cells to secrete tumor antigen-specific antibodies *in vivo*. Approaches targeting shared mutations serve as excellent ‘off-the-shelf’ options which can be used for larger groups of patients simultaneously. Cancer vaccines based on shared mutations are also clinically important, provided the patients’ HLA profiles are matched to the epitope binding characteristics. Antibodies derived from tumor-infiltrating B-cells or from peripheral blood B-cells targeting surface-bound shared neoantigens may mediating cellular cytotoxicity and aid in the development of CAR T-cells. Gene therapy to correct shared driver mutations may promote tumor susceptibility to immune attack. Immune checkpoint blockade has been placed between the two domains as its clinical activity targets both private and shared mutated targets. Similarly, NK, TCR γδ T-cells and possibly NKT T-cells or MAIT-cells may be instrumental in patients presenting with private and/or shared HLA pathway mutations and can be derived from allogeneic sources for treatment.

Tumor mutational burden (TMB) is a key factor in determining the response of patients with cancer to immunotherapy with immune checkpoint inhibitors (anti-programmed cell death 1 [PD-1] or anti-cytotoxic T lymphocyte-associated antigen 4 [CTLA-4]) (2–7). The ‘mutanome’, the summary of mutations developing over the course of disease is unique from one patient to another, thus making the TMB a unique biological signature comprising of druggable targets and epitopes to elicit anti-cancer immune responses. Alexandrov and colleagues elegantly showed that varying degrees of TMB are associated with different cancer types, and that disease-specific mutational signatures may either be widespread (e.g. melanoma and lung cancer) or restricted (e.g. pancreatic cancer) to certain parts of the genome – thus influencing the number of mutant genes and inevitably the availability and immunogenicity of neoantigens (8). A large proportion of favorable clinical responses rely on a rich reservoir of tumor-infiltrating lymphocytes (TIL) as well as circulating tumor-directed T-cells and, therefore, TCRs which recognize neoepitopes presented by human leukocyte antigen (HLA) molecules on tumor cells (9–19). The number of mutations which are identified through bioinformatics directly influence the repertoire size of immunogenic targets that may induce T-cell responses and potentially anti-tumor directed T-cell responses (**Figure 1**). Although companion diagnostics for PD-1, programmed death-ligand 1 (PD-L1) and CTLA-4 are actively used prior to initiating immunotherapy to confirm expression in tumor tissue samples, mutations in the HLA pathways may often be overlooked – which will impair or abolish productive anti-cancer directed cellular immune responses. In addition, other immunologically relevant mutations or natural variations which may inherently affect immune function and T-cell responses deserve equal attention if these factors influence the quality and quantity of anti-cancer directed immune responses. The TMB is still considered a key factor in predicting clinical responsiveness or to gauge the possibility of the immune system to productively react against cancer cells. Yet the TMB represents only the substrate of potential immune reactivity and the immune system is not objectively considered and analyzed. The TMB is therefore increasingly viewed as an important yet ‘imperfect’ surrogate marker for clinical responsiveness and the corresponding elements in orchestrating a cellular immune response, namely the MHC genetic background as well as the T-cell receptor repertoire capable of reacting to potential cancer neopitopes, are now considered to be analyzed as well to gauge for immune response analysis (20). The nature and the histological location of T-cells that serve to functionally test for immune recognition of neoepitopes are therefore also considered in this review. We will also highlight in this review relevant findings from clinical and translational studies pertaining to personalized cancer immunotherapy. We discuss HLA mutations in tumor lesions from patients with cancer and discuss how this information is necessary for designing personalized immunotherapy clinical trials. Finally, we propose the combined use of well-established techniques such as immunohistochemistry (IHC) and flow cytometry in conjunction with next-generation sequencing methods to assist in making better informed clinical decisions for treatment regimens, a concept that has been implemented at the ImmunoSurgery Unit and Anatomic Pathology Clinical Service at the Champalimaud Centre for the Unknown (CCU) and the Champalimaud Clinical Centre (CCC) in Lisbon, Portugal (**Figure 2**).

## Next-Generation Sequencing: The Fuel of Precision Oncology

Advances in next-generation sequencing (NGS) techniques such as WES and RNA-seq form the bedrock of personalized precision medicine in neoantigen-directed immuno-oncology (22, 23). Immunoediting leading to neoantigen generation and turnover in the tumor microenvironment (TME) influencing T-cell infiltration and survival in patients with advanced cancer (24–26). This also goes hand-in-hand with the MHC background of the patient as well as the capacity to present the ‘best’ neoepitope candidates to evoke meaningful and clinically beneficial T-cell responses (27–29). Importantly, juxtaposition of tumor-specific T-cells to the tumor cells themselves provides promising prognosis, suggesting that the local ‘cell-cell’ interaction between neoepitope-specific lymphocytes and tumor cells is clinically beneficial and desirable (30). Treatments affecting the activity of cancer-associated fibroblasts (CAF) or tumor-associated macrophages (TAM) (e.g. monoclonal antibodies such as anti-CD47 or anti-CD40) redirect T-cells to these nominal target cells which appear to be associated with improved anti-tumor responses in a clinical setting (31–36). Furthermore, evolution of the neoantigen landscape under treatment pressure, such as standard chemotherapy, immune checkpoint blockade or active cellular therapy/adoptive cell transfer (ACT) is an essential determinant of how patient immune response patterns are modulated and change over time (37, 38). In line with this, neoantigen fitness – the propensity of mutated host targets which differ significantly enough from the wildtype form to be able to produce a biologically meaningful anti-tumor response, further to their HLA-binding strength – can be mathematically modeled to predict survival dynamics of patients and aid in the selection of promising neoepitope candidates for immunotherapy protocols (39). The fitness of a (cancer) cell clone is defined by several factors, e.g. the recognition potential of the (immunodominant) neoepitopes by nominal anti-cancer directed TCRs that will aid to estimate the future size of the cancer cell population. ‘Immuno-dominance’ can be gauged by comparing the affinity of the wildtype and the corresponding mutant candidate target epitope that would impose selective pressure on the clonal pool of available TCRs that recognize the MHC-peptide complex.

Clinically, TIL therapy targeting individual neoepitopes has been proven to be successful, with the capacity to promote durable anti-tumor responses in patients with solid tumors (17, 40). Clinical responses appear to be associated with the frequency of neoepitope-specific T-cells in the T-cell product (40). Mutant KRAS-directed TIL and TCR transfer therapy has also shown great clinical promise, albeit in an HLA allele-dependent manner (41). In addition to neoantigens, non-coding regions of the cancer genome giving rise to previously undefined, non-mutated peptides with immunogenic properties can also be mined for, using NGS strategies (42), as well as peptides resulting from novel gene fusions (43). This pattern may differ from patient to patient, necessitating the use of *in-silico* analyses to select matching HLA-epitope sets for a personalized therapy protocol. Thus, private and shared neoantigens as well as hitherto unknown immunogenic peptides can trigger beneficial clinical responses in patients with advanced cancer (16, 44, 45).

NGS readouts combined with recent advancements in immune-based analysis of patient-derived tumor and blood samples are able to provide a wealth of information concerning the presence of dynamics of cancer-specific T-cells suitable for immunotherapy development or for immuno-monitoring following treatment, including neoepitope specificity and TCR tracking (14, 18, 46–49). Neoepitope screening has enabled the identification of private mutation-directed TIL from pancreatic cancer (18, 45, 50) and glioblastoma (14) demonstrating that tumor histologies previously considered poorly immunogenic may also contain a broad repertoire of neoantigen-reactive immune cells (11, 16, 51–53). Specific neoepitopes involved in eliciting productive immune responses that promote tumor regression either by engaging cellular cytotoxicity or by cytokine production (e.g. IFN-γ) are therefore particularly attractive for developing personalized therapies within the framework of precision cancer medicine (54, 55). However, there is also a need to identify target neoepitopes which are most likely to induce regulatory T-cell responses among TIL to the effect of dampening productive anti-tumor reactivity in patients (56).

Druggable mutations (e.g. those associated with ROS1, ALK, tropomycin receptor kinase [TRK] and NTRK1/2/3 chromosomal fusions) which have been implicated to be responsible for clinical responses in pediatric central nervous system (CNS) malignancies provide a roadmap for how NGS is able to support precision oncology based on selected small molecules (i.e. Entrectinib and Larotrectinib) (57). Hitherto unknown mutational events can be captured *via* NGS, possibly expanding the use of existing targeted cancer drugs and, in addition, newly devised immunotherapeutic strategies. These probable candidates can be tested for T-cell and antibody reactivity *in vitro*, and positive results can then be followed up with more detailed analysis to enable the formulation of personalized cancer vaccines (PCVs) or cellular immunotherapy development (chimeric antigen receptor T-cells [CAR-T], TCR transfer, ACT of TIL and/or memory B-cells), paving the way for combination therapies, e.g. with tyrosine kinase inhibitors (TKIs) and immune-based interventions.

## Personalized Cancer Vaccines

Building on the therapeutic value of targeting cancer-associated mutations, mutation-directed cancer immunotherapy based on PCVs represent a highly specialized approach to induce clinically relevant and specifically tailored anti-tumor immune responses in patients with advanced malignancies (54, 58, 59). A central point is whether epitopes can be presented by HLA class I or II molecules based on their fitting into the epitope-binding groove and be tailored *in silico*, or whether natural processing by antigen-presenting cells (APCs) in the host or dendritic cell (DC)-based vaccines would be more advantageous (e.g. if antigens were delivered as “long peptides”) (60–62), or in a vectored format (e.g. genetically-reengineered viruses and bacteria) (63–68). A carefully selected panel of private and shared cancer-related mutations (e.g. common driver mutations in genes such as *KRAS*, *SMAD4*, *TP53*) identified by WES that bind to the HLA class I and II restriction elements of the patient constitute the formulation of some PCVs (27, 69–72). New research based on high-throughput NGS data shows that the hydrophobicity of predicted neoepitopes could, in part, determine better HLA-binding capacity (28). Longer peptide sequences are likely to contain both HLA class I and class II peptides and would, therefore, activate tumor-directed CD8^+^ and CD4^+^ T-cells facilitated by cross presentation of antigens in antigen-presenting cells (i.e. DC, macrophages, B-cells as well as tumor cells) (58, 73). PCV constituent peptides may also be used as lead molecules to construct HLA tetramers or as T-cell stimulants to screen for the presence of neoantigen-specific TCRs in blood samples of patients with cancer (16, 44, 74).

A number of trials to test neoantigen-based PCVs in patients with advanced cancer – including pancreatic cancer – have been registered (58, 59, 75). PCV strategies which have been clinically evaluated are based on direct delivery of messenger RNA (mRNA) sequences of private neoepitopes to the lymph nodes (76), dendritic cells loaded with the patient’s tumor lysate, private mutated peptides (neoantigens) (12, 71, 77–80) or clinical-grade neoepitope peptide sequences injected alongside a strong adjuvant or immunostimulant (i.e. poly-ICLC) (69, 72). Montanide^®^, which is based on antigens from *Mycobacterium tuberculosis* (81–84), and QS-21, extract derived from the soap bark tree *Quillaja saponaria* (85, 86), are also candidates for use as adjuvants in PCVs based on previous clinical experience. Hu and colleagues have comprehensively summarized and elaborated on the current landscape in PCV development (23).

Pertaining to the clinical testing of cell-free, neoepitope-based peptide vaccines, Keskin and colleagues recently reported a phase 1b PCV clinical trial in eight patients with glioblastoma, where specific CD4^+^ T-cell responses to a mutation-bearing sequence from Rho GTPase Activating Protein 35 (ARHGAP35), which is naturally processed and presented to the immune system, were demonstrated in one patient (72). Furthermore, the authors also noted increased T-cell infiltration into the tumor – concomitant with neoantigen-specific T-cell in peripheral blood – following PCV administration. In a previous study, the authors had treated six patients with advanced melanoma and showed, that despite including HLA class I-binding peptides (for CD8^+^ T-cell recognition) in the vaccine design, superior CD4^+^ T-cell responses directed against the patients’ neoantigens was observed (69). There is until this point not a convincing biological model for the observation that presumed MHC class I binding peptides (87), delivered as 9mers, induce rather CD4^+^ T-cell responses as compared to CD8^+^ T-cells; an observation that has been found to be true in several vaccination studies using tumor-associated mutant targets (69). In a different study, in patients with glioblastoma, CD4^+^ T-cell responses were dominant in the case if mutant (nested) MHC class I-restricted epitopes were used for vaccination. None of the mutant epitopes elicited solely a CD8^+^ T-cell response (although MHC class I epitope clusters were used), yet rather immune responses restricted by CD4^+^ T-cells alone or by CD4^+^ and CD8^+^ T-cells were detected (88).

PCV-induced immune responses can also be enhanced with anti-PD-1 therapy (62, 69, 76). The TCR repertoire identified in clinically relevant and successful immune checkpoint therapy responses is associated with different anatomical compartments (89) as well as distinct T-cell markers in antigen-specific T-cells including CD103^+^ T-cells (90) or – more recently with an stem-cell like CD8^+^CD69^-^CD39^-^ phenotype in TIL that is strongly associated with clinical responses (90).

The rationale for increasing clinical responsiveness to PCV with checkpoint inhibitors would be to mobilize mutation-specific T-cells and PD-1^+^ B-cell populations specific for cancer mutations (91–93). A different approach which may improve fine-tuning of anti-tumor responses following PCV treatment is the removal of non-productive inflammation caused by interleukin 6 (IL-6). Generally important for priming T-cell responses (94), IL-6 is a pleiotropic cytokine implicated in the pathogenesis of several cancer histologies, particularly gastrointestinal malignancies including pancreatic cancer and colorectal cancer (95, 96), one of the key factors being the suppression of productive immune responses in the TME (97–101). Treatments targeting IL-6 are approved for clinical use in patients with rheumatoid arthritis and Castleman’s disease (102), although their use in patients with advanced cancer has yet to be fully realized despite promising results from preclinical models of solid tumors (94). Furthermore, IL-6 is among the cytokines released in large amounts following T-cell therapies for cancer (i.e., adoptive cell transfer ACT or CAR-T cell therapy) (103). IL-6 can lead to upregulation of PD-1 and immune exhaustion (104), while promoting interleukin 17 (IL-17) production – which can be a disadvantage in patients with cancer to subduing T-cell activity and augmenting tumor cell proliferation (105–107). Combined targeting of IL-6 and the PD-1/PD-L1 axis has shown reversal of immunosuppression in the TME leading to immune activation and tumor rejection in murine models of human cancer (108, 109). Other approaches in increasing the amenability of the TME to therapeutic intervention are to target the extracellular matrix and tumor stroma which provide scaffold support for the cancer cells (110, 111) and TAMs which have a pro-tumor effect in the TME (112).

Recent peptide vaccine trials show the complex neoepitope selection process and validation process – and underline also the need for a more harmonized approach that will enable to compare results across different studies to gauge T-cell responses against the immunizing peptides. In a clinical study for patients with melanoma (69), WES of the tumor was conducted, validated by RNA-Seq and mutant (tumor) peptides were selected based on the patient’s HLA-A and -B alleles followed by production of long-peptides representing up to 20 neoepitopes per patient. The ‘private vaccine’ was administered with an TLR3 adjuvant (poly-ICLC). MHC class I binding was predicted *via* NetMHCpan v2.4 and neoepitopes were selected with a hierarchy of criteria: i) frameshift mutations where the algorithm predicted binding, ii) single nucleotide variants (SNVs) where the algorithm predicted binding due to the mutation being in an anchor residue, iii) SNVs where the algorithm predicted binding due to the mutation being in residues other than anchor residues, iv) frameshift mutations where the algorithm did not predict binding and v) SNVs where the algorithm predicted low binding. In addition to the criteria listed above, oncogenes were prioritized and biochemical constraints concerning peptide synthesis were considered. ‘Long peptides’ allow for antigen processing and presentation for both CD4^+^ and CD8^+^ T-cells. The immunological readout addressed biological and clinically relevant questions like the frequency of peptide-specific T-cells (*in vitro*) upon re-stimulation assays using peripheral blood mononuclear cell (PBMCs) as immune effector cells showing that T-cell responses could indeed be induced against each individual vaccine target antigen. This point has practical implications: target antigen peptides were screened for T-cell reactivity and the biological readout is usually IFN-*γ* production. Non detectable IFN-*γ* production could imply that – assuming that the candidate peptide shows MHC binding – the frequency of T-cells directed against the candidate epitope is either low or that there are no peptide specific TCRs available in an individual patient. Low T-cell frequencies specific for a candidate peptide implies that these T-cells have not yet been expanded *in vivo*. Such a candidate peptide may represent a viable neoepitope for vaccination or T-cell expansion particularly if it is able to recruit T-cells from a stem cell pool with promising expansion potential and anti-cancer directed immune effector functions (90). Different peptide testing formats were used to gauge for the immune T-cell reactivity readout: a) peptides, b) minigenes (that allow the use of surrogate antigen presenting cells in order to test whether the peptides are naturally processed and presented, assuming that these minigenes are similarly processed as compared to the wildtype target tumor antigen), c) autologous tumor cells in order to test whether tumor cells are recognized by peptide-expanded T-cells since antigen processing and presentation may be different in tumor cells as compared to non-transformed cells as reviewed in Vigneron (113). This example shows the critical steps in the workflow and decision making process for which antigens should be selected (oncogenes, frameshift mutations), the format for vaccination (long peptides), the nature of the adjuvant and the question whether candidate peptide-reactive T-cells recognize naturally processed epitopes, the nature of the immune response, i.e., cytokine production (using intracellular cytokine staining to gauge for polyfunctional T-cells), a CD107a induction assay (to gauge for cytotoxicity), as well as a direct enumeration of MHC-specific T-cells using soluble MHC-peptide complexes. The list of different assays above reflects that peptide recognition may be tested positive in a specific biological readout (e.g. cytokine production), but not in another (e.g. cytotoxicity). Of practical interest is that MHC-class II peptide-tetramer guided enumeration often underestimates antigen-reactive CD4^+^ T-cell numbers since the MHC-peptide interaction is fixed. In contrast, the incubation time of candidate test peptides with PBMCs is usually a few hours (and takes place at a different temperature) – allowing to accommodate a more ‘promiscuous’ binding of peptide species to several MHC class II alleles. Analysis of peptide-reactive T-cells with soluble MHC-peptide complexes provides unbiased enumeration of MHC-peptide reactive T-cells since it enables *ex vivo* analysis without the need of *in vivo* T-cell expansion, it enables – *via* the co-staining of T-cell differentiation and activation markers (that define in which T-cell compartment the antigen-specific T-cells reside) – to link the *ex vivo* analysis of antigen-specific T-cells with T-cell homing, differentiation, maturation or functional markers associated with cytokine production. This is clinically relevant since tumor-reactive T-cells that – upon adoptive transfer – lead to clinically relevant response reside preferentially in the central memory T-cell subset and/or exhibit distinct activation (CD69^-^CD39^-^) phenotypes (90). Examples of two vaccination trials with peptides identified from glioblastomas addressed different, clinically relevant points, namely whether the presence of (candidate) antigen specific T-cells prior to vaccination would predict successful vaccination outcomes and whether the nature (mutant versus non-mutant targets) would make a difference in regard to T-cells expansion (88). This first, rather complex, clinical trial, was conducted using an ‘off-the-shelf’ cocktail of non-mutant peptides of glioblastoma-associated antigens targeting HLA-A2 and HLA-A24-positive patients, plus candidate ‘private peptides’, either from mutant or non-mutant ‘private’ glioblastoma targets. Key observations were that i) binding of some candidate peptides to MHC molecules was confirmed by mass spectrometry, i.e., these peptides were found to be naturally processed and presented, ii) mass spectrometry allowed an unbiased analysis of the peptide repertoire displayed by cancer cells, within the detection limits, yet requires approximately 10e^7^ tumor cells for analysis (114), iii) MHC-class I restricted CD8^+^ T-cell responses, usually residing in precursor T-cells, to non-mutant epitopes prior to vaccination predicted successful T-cell responses after vaccination, iv) some peptide vaccine-induced T-cells recognized naturally processed and presented epitopes on the patients’ autologous tumor cells, v) vaccination with CD8^+^ T-cell epitopes induced CD4^+^ T-cell responses, iv) some mutant vaccine epitope resulted in T-cells recognizing wildtype and mutant target antigens, v) none of the mutant epitopes evoked an exclusive CD8^+^ T-cell response, but rather CD4^+^, or T-cell responses in CD4^+^ and in CD8^+^ T-cells, vi) T-cells expanded from glioblastoma tissue harvested prior to vaccination did not contain T-cells responding to the selected candidate target epitopes, vii) no preferential expansion of T-cells using mutant epitopes as compared to non-mutant epitopes. A different clinical trial, also in patients with glioblastoma, showed that T-cells induced by vaccination infiltrated into tumor lesions after peptide vaccination (115). This demonstration is clinically very relevant since only a few studies were able to demonstrate that T-cell clones elicited by peptide vaccination – reacting against the immunizing peptide – would then hone to the patient’s tumor and aid to mediate tumor regression. This argues that it is necessary to obtain biopsies in progressing and regressing tumor lesions from patients with cancer who undergo peptide-based vaccination. Of particularly clinical relevance is i) that patients who received corticosteroids (which most patients with glioblastoma receive) during vaccine-priming failed to generate IFN-γ production to vaccine peptides, ii) some vaccine-peptides induced T-cells reacting against minigenes (coding for these targets, expressed as transgenes into surrogate recipient targets cells), but not to tumor cells, iii) that a round of *in vitro* stimulation was needed in order to detect antigen-specific T-cells, reflecting most likely low antigen-specific T-cell frequency, iv) peptide antigen-driven expansion *in vitro* and subsequent single cell PCR sequencing allowed to link TCR usage to peptide specificity. The identification of the unique peptide-specific TCR CDR3 motif allowed to ‘trace back’ the antigen specific T-cells to time points prior to vaccination (and post-vaccination samples) to the tumor sample used to identify the private mutations, as well as to post-vaccination tumor samples in case of tumor recurrence. Some mutant peptide specific TCRs were not detected prior to vaccination in PBMCs (which represent only 2% of the entire lymphocyte pool), nor have they been found in the tumor specimen used for mutational analysis, yet they were detectable in the tumor recurrence, an observation that was also observed in a rather more anatomically accessible basal cell cancer study (116). Such antigen-specific T-cells are mediating anti-tumor responses and their detection allow therefore a biologically relevant clue how neoepitope-specific T-cell therapies could be improved: Anti-cancer-directed T-cells after checkpoint inhibitor therapy were not ‘rescued’ or epigenetically rewired in response to checkpoint inhibitor therapies (117), yet rather ‘new’ T-cells were able to access the tumor site upon checkpoint inhibitor treatment. This phenomenon was dubbed ‘clonal replacement’ and would also support the notion that peptide-induced T-cells are able to access cancer lesions after vaccination (116). These observations are reminiscent of anti-cancer directed vaccine trials almost 2 decades ago. Although vaccination with (non-mutant) tumor associated antigens resulted in clinically relevant responses, the regressing tumor lesions showed ‘spontaneous’ anti-cancer immune reactivity, yet anti-cancer vaccine responses were not detected in the regressing cancer lesions suggesting that tumor vaccination aids to reinvigorate immune responses to private cancer antigens – and that a competent TCR is a prerequisite to achieve clinically meaningful T-cell responses (118). The practical consequence of these observations is to perform 2 or 4 mm needle biopsies in accessible tumor lesions that would allow to gauge for deep TCR sequence analysis and to trace mutant-epitope specific T-cell clones. In addition, both studies targeting glioblastoma epitopes showed that mutant peptide epitopes favored expansion of cytotoxic CD4^+^ T-cells (115) and even if peptides were used to target CD8^+^ T-cells, peptide antigen-specific CD4^+^ T-cell expansion was observed in both studies (88). These and other clinical trial data were recently excellently reviewed addressing the clinical utility of neoantigen identification, peptide processing and MHC presentation of candidate epitope targets for rational vaccine design (119).

## Immune Function and Personalized Immunotherapy

Personalized immunotherapy is based on the capacity of the immune system to recognize, to be activated, to clonally expand and ultimately to kill off or to contain cancer cells. This involves several biological pathways, including the recognition and response to danger-associated molecular patterns (DAMPs) by cognate receptors present on the surface of APCs, T-cells as well as parenchymal cells (120). DAMPs are either released into the environment [e.g. high-mobility group box 1 (HMGB1), adenosine triphosphate, calreticulin; reviewed in (121)] or they can be cell-bound (Fas ligand [FasL] (122), heat shock proteins (123), MHC class I polypeptide-related proteins A/B [MICA/B]) (124, 125), and upon encountering the suitable receptor, elicit a signaling program resulting in an pro-inflammatory response. Although DAMPs can lead to non-productive inflammation resulting in organ damage, they play nevertheless an essential role in promoting cancer-directed immune responses and form an integral component of personalized immunotherapy strategies (126). Equally important is the epigenetic regulation of DAMPs which aid to the successful orchestration of innate and adaptive immune responses in PCV trials and clinically relevant immune responses (127). The use of ‘in-built’, molecularly defined adjuvants such as non-coding RNA may also augment the capacity of immune cells to orchestrate a potent anti-tumor immune responses *in vivo*, e.g. by activating the RNA sensing molecule retinoic acid-inducible gene I (RIG-1) (128). Indeed, RIG-I and a related intracellular RNA-sensing molecular melanoma-differentiation factor 5 (MDA-5) have been discussed as potential players in amplifying anti-tumor immune responses following recognition of cancer-associated RNA structures (128, 129). Other players in immunosurveillance are the toll-like receptors (TLR) 3 and 7, that are also involved in recognizing RNA derived from pathogens, with TLR3-mediated immune activation playing an essential role in the clinically relevant immunogenicity of poly-ICLC, a synthetic, double-stranded RNA-based polymer used as an adjuvant in the formulation of personalized cancer vaccines (69, 72, 76).

In a similar manner, different microRNA species are likely to be involved in immunomodulation and enhancement of local immune surveillance in cancer lesions (13, 130–132). Although the immunosuppressive TME may result in the downregulation of microRNA species, an unbiased identification of promising microRNAs and non-coding RNA sequences is possible *via* NGS and would allow to test synthetically produced RNA sequences as components in immuno-stimulation in PCV studies. MicroRNA species may also subdue expression of neoantigen-encoding genes, identifying microRNA using RNA-seq will therefore reveal additional layers of genetic regulation interfering with optimal anti-tumor immune responses, yet it also opens new molecularly defined ways to optimize anti-cancer directed therapies in a more evidence-based fashion.

The stimulator of interferon genes (STING) pathway augments as well anti-tumor cellular immune responses (previously reviewed (133–135)), including potent B-cell activation and antibody production (136). STING is encoded by the TMEM173 gene in humans and acts as an intracellular DNA-sensing molecule (thus a pattern recognition receptor [PRR]) requiring cyclic guanosine monophosphate–adenosine monophosphate (GMP-AMP) synthase recognition of cytosolic DNA involved in expression of type I IFN-regulated pro-inflammatory genes. Activation of STING leads to transcription of IFN-stimulated response elements (ISREs) *via* TANK Binding Kinase 1 (TBK1) activation and interferon regulatory factor 3 (IRF3) localization into the nucleus to initiate gene transcription. Several clinical trials are underway, testing STING pathway agonists to induce anti-tumor immune responses in patients with cancer. A three-prime repair exonuclease 1 (TREX1) expression is involved in dampening STING-mediated immune activation by eliminating damaged DNA from the cytosol (135). However, it is possible that TREX1 mutations in patients with cancer may instead increase immune activation in cancer cell along with STING stimulation. Mutations in the STING pathway have been reported in patients with colorectal cancer, where STING-deficient cancer cells were unable to produce interleukin 1β (IL-1β) in response to DNA damage (137). Preclinical research showed that STING-dependent immune activation was able to enhance neoantigen vaccine responses and changed favorably the TME immune profile (138–140). Thus, primary and secondary immunodeficiencies defined by NGS can be identified molecularly and should supplement the NGS information obtained from cancer cells. STING variants may be naturally occurring genetic aberrations (e.g. silencing mutations in TLRs or receptors recognizing DAMPs), be associated with infections (e.g. human immunodeficiency virus [HIV]) or with immunosuppressive therapies (e.g. solid organ/stem cell transplantation and therapies used for patients with autoimmune diseases (120, 141–144)). NGS readouts allow also to visualize the mutational status of the STING pathway – among other immune-activating genes – in validating neoantigen-directed immune responses when designing PCVs and transgenic TCR-based cancer treatments. A comprehensive panel of mutations and natural variants in key molecules orchestrating the quality and quantity of anti-cancer directed immune responses is screened within the cancer NGS analysis in the ImmunoSurgery unit at the Champalimaud Foundation (see below) in order to better define immunological landscape of local and systemic immune responses that may influence immunotherapeutic strategies.

## Antigen Processing and Presentation Machinery Mutations in the Context of Immunotherapy

**Figure 3:**
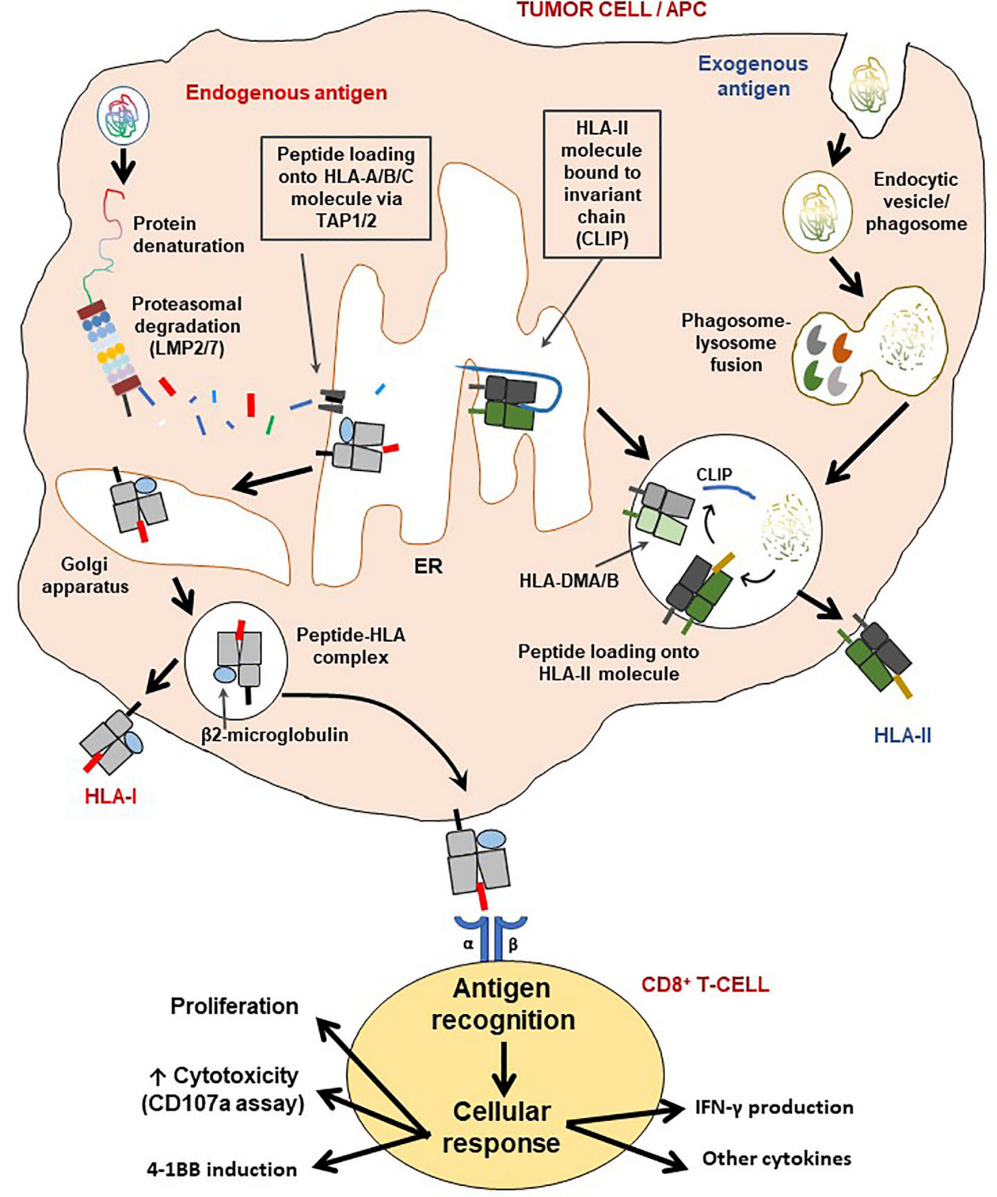
Schematic representation of the HLA class I and II pathways and T-cell activation. The HLA class I pathway is also known as the intrinsic pathway as it processes and presents endogenous antigens while antigens derived from the extracellular environment are processed and presented *via* the HLA class II (extrinsic) pathway. LMP2/7 are immunoproteasome subunits necessary for generating short epitopes (7-11 amino acids along), which are then loaded on the HLA class I molecule for presentation to CD8^+^ T-cells. The β2-microglobulin (β2M) is critical for the assembly and stable expression of HLA class I-peptide complexes on the cell surface. On the other hand, HLA class II molecules first exist with the class II-associated invariant chain (CLIP) for stability, which is then removed with assistance from the HLA-DMA/B complex, for loading of CD4^+^ T-cell epitopes generated *via* lysosomal degradation. Processed antigens are then presented by either HLA-II (extrinsic pathway) or HLA-I (intrinsic pathway), to T-cells to initiate an immune synapse followed by activation of the latter. Indeed, as a result of cognate antigen recognition, T-cells may produce one or a combination of effects: i) cellular proliferation (also involves IL-2), ii) increase in cytotoxicity (may be measured by surface CD107a induction assay), iii) induction of 4-1BB expression and/or iv) production of cytokines, such as IFN-γ, TNF-α, IL-2, IL-17c.

The antigen presentation machinery (APM), mainly constituted by the HLA class I and class II antigen processing and presentation pathway, are central to immune recognition and immune surveillance. While the HLA class I pathway generally processes and presents endogenous antigens derived from intracellular pathogens or autoantigens (such as neoantigens in cancer), the HLA class II pathway processes and presents exogenous antigens, which could be host- or pathogen-derived. CD8^+^ T-cells are HLA class I restricted while CD4^+^ T-cells recognize HLA class II epitopes (145). While all cells of the body (except erythrocytes) constitutively express the HLA class I pathway (except in the CNS, where MHC class I is downregulated); the HLA class II pathway can be activated in the presence of IFN-γ *via* transcription of the class II transactivator, thus underlining the need for IFN-γ in the TME (146). The standard APM in human cells is shown in **Figure 3**.

The antigen processing and presentation machinery is of major importance in immunosurveillance, as mutations occurring in the HLA class I and class II pathways bear great significance to cancer immunotherapy. Loss of MHC molecules may lead to immune-escape which may entail the failure of clinically relevant immune surveillance, loss of individual MHC class I loci in cancer lesions prohibits targeted therapy using PCV, since the identification of allelic losses limits naturally the choice of peptides to be used in a PCV. Thus, detailed analysis of the HLA haplotypes (HLA-A/B/C), is a prerequisite in selecting the ‘best-fitting’ neoepitopes in the design of personalized cancer vaccines as well as TCR-dependent T-cell therapies (28, 71, 147, 148). Downregulation of and loss-of-function mutations in the HLA class I and II pathways abrogate or dampen immune recognition of tumor cells *in vivo* (149–155), which goes along with TME evolution in response to immune activation (26, 156). Components of the HLA class I and class II pathways, if affected by genetic aberrations, may lead to ‘tumor antigen loss variants’ (thus the inability to process and present immunologically viable neoantigens) (157, 158). The ‘hyper progression’ effect described in patients treated with checkpoint inhibitors may, in fact, reflect an HLA loss *in vivo* while subtle MHC mutations may also have a similar deleterious effect on immune recognition by TIL if such mutations affect the nature and diversity of the peptide repertoire loaded onto the nominal MHC molecule. In accordance, mutations in the canonical HLA class I pathway (HLA-A/B/C) and its associated components in patients with cancer have been previously described, e.g. transporter associated with Antigen Processing 1/2 (TAP1/2), latent membrane protein 2/7 (LMP2/7) and β2-microglobulin (151, 159–164). Some tumor cells may also contain alternate splice forms of tapasin that can alter the repertoire of peptides loaded into the MHC class I antigen presentation pathway (165), mutations in the MHC class II antigen processing and presentation pathway have also been described (166, 167), yet they are not reported as frequently as molecular defects in the HLA class I pathway. There has been however much attention given to MHC class II expression in various cancer types such as colorectal (168), cervical (169), lung (170), breast (171), melanoma (172) and pancreatic cancer (173), pointing also to the significance of CD4^+^-mediated anti-tumor responses (174, 175). Components associated with HLA class II peptide loading (the invariant chain [CLIP]), as well as the “peptide editors”, HLA-DMA/DMB/DOA/DOB also play a significant role in producing meaningful CD4^+^ T-cell responses (153, 168, 169) and are needed to present tumor antigen derived epitopes to CD4^+^ T-cells either by cancer cells directly or APCs in the TME cross-presenting tumor-associated antigens. This is of clinical relevance since PCV enriched for CD8^+^ T-cell epitopes tend to induce target-specific CD4^+^ T-cell responses as discussed above. The loss of HLA class I expression and therefore subsequent CD8^+^ T-cell responses in a patient with pancreatic cancer has been observed to be compensated with HLA class II-restricted CD4^+^ T-cells (with cytokine production and cytolytic activity) (18) arguing that a molecular and immuno-histological examination of cancer lesions should include the MHC class I as well as the MHC class II antigen processing and presentation pathways that can be subject to therapeutic modulations, e.g. using HDAC inhibitors.

## A Role for Unconventional Neoantigen Presentation in Cancer?

Reduced expression of HLA-E (non-canonical HLA class I) has been linked to increased survival of patients with ovarian cancer (176), while it may also inhibit CD8^+^ TIL activity. MICA and MICB are expressed in the gastrointestinal epithelium, thus with relevance for metastatic GI cancers, e.g. colorectal or pancreatic cancer. Both MICA and MICB molecules are stress induced and bind to NKG2D in T-cell engagement, which could lead to activation of NK cells (or – not mutually exclusive – activation of TCR *γ*δ^+^ T-cells) instead of the TCR αβ^+^ T-cells. HLA-G is yet another non-classical HLA class I member, whose expression is associated with a poor prognosis for patients with cancer, including patients with glioblastoma, colorectal and pancreatic cancer (177). Furthermore, HLA-G can be found in soluble form in blood while also secreted in exosomes; it can also be readily detected in IHC – thus making it feasible also for immunodiagnostics. Non-classical HLA molecules should also be considered in the development of anti-tumor directed vaccination – and future preclinical developments will target the identification of tumor-associated antigens presented by non-classical MHC-molecules to anti-cancer directed immune cells including the non-classical Major histocompatibility complex class I-related (MR1) molecule (178).

The presentation of non-peptide antigens by the cluster of differentiation 1 (CD1) family of molecules (a, b, c, d) – which is related to HLA class I – leads to activation of unconventional T-cell subsets, such as natural killer T-cells (NKT) (e.g. lipid antigens) and TCR γδ T-cells (e.g. phosphoantigens), with CD1d being the most prominent member, expressed on epithelial cells – and epithelial cancer cells. Expression of CD1 molecules in cancer are associated with different clinical outcomes while associated with poor prognosis in renal cell carcinoma (179), CD1d promotes NKT-mediated cytolysis of cancer cells in lung adenocarcinoma (180). A similar scenario exists for multiple myeloma and B-cell lymphoma, since CD1d is downregulated and associated with poor outcomes in contrast to higher CD1d expression levels in PBMCs from healthy individuals (181).

Nevertheless, neoantigen classes (and neoepitopes thereof) are limited to protein-based structures at this juncture due to their recognition by conventional HLA/TCR interactions involving CD4^+^ and CD8^+^ T-cells (13, 38, 182). More research is necessary to project a more concise picture of the role of non-peptide entities, e.g. lipids and carbohydrates bearing cancer-associated molecular abnormalities in augmenting productive immune responses in patients. For example, overexpressed or aberrantly glycosylated carbohydrates (e.g. gangliosides) is now hailed as a CAR-T target to treat pediatric patients with solid tumors (183). Also, the recognition of several pathogen-derived carbohydrate structures by conventional T-cells has been previously reviewed (184). The clinical studies associated with such therapeutic approaches could provide a template for precision oncology methods e.g. investigating which sugar moieties harboring abnormalities would be recognized by specific T-cell subsets using NGS and immunological assays.

## Accounting for Lymphocyte Classes in Precision Immunotherapy Design

Multimodal studies have shown that the local immune landscape as well as neoantigen expression are quintessential parameters in determining and driving clinical responses in patients with cancer (13, 37, 185–188). Data from translational and clinical cancer immunotherapy studies collectively advocate for the development of ‘composite lymphocyte grafts’ comprising several immune-cell types interacting with a broad array of neoantigen profiles and subsequently a diverse set of effector functions with the unified aim to minimize disease progression, while eliminating existing cancer cell reservoirs in the patient (189, 190). Tumor infiltrating immune cells as well as tissue resident cells contribute to shape the immunological milieu, which is worthwhile to consider in precision immunotherapy protocols.

T-cells can be harvested and expanded for immunotherapy mainly from cancer tissue (TILs, inflamed tissue-derived cells), and/or PBMCs (11, 19, 44, 47, 191–193), cells from pleural effusions may also serve to isolate tumor-reactive T-cells (194) as a possible T-cell source, as well as immune cells from bronchoalveolar lavage (195), cerebrospinal fluid (196) or bone marrow aspirates (197). This biological material is a yet rather untapped source for future assessments in T-cell immunotherapy trials.

**Figure 4:**
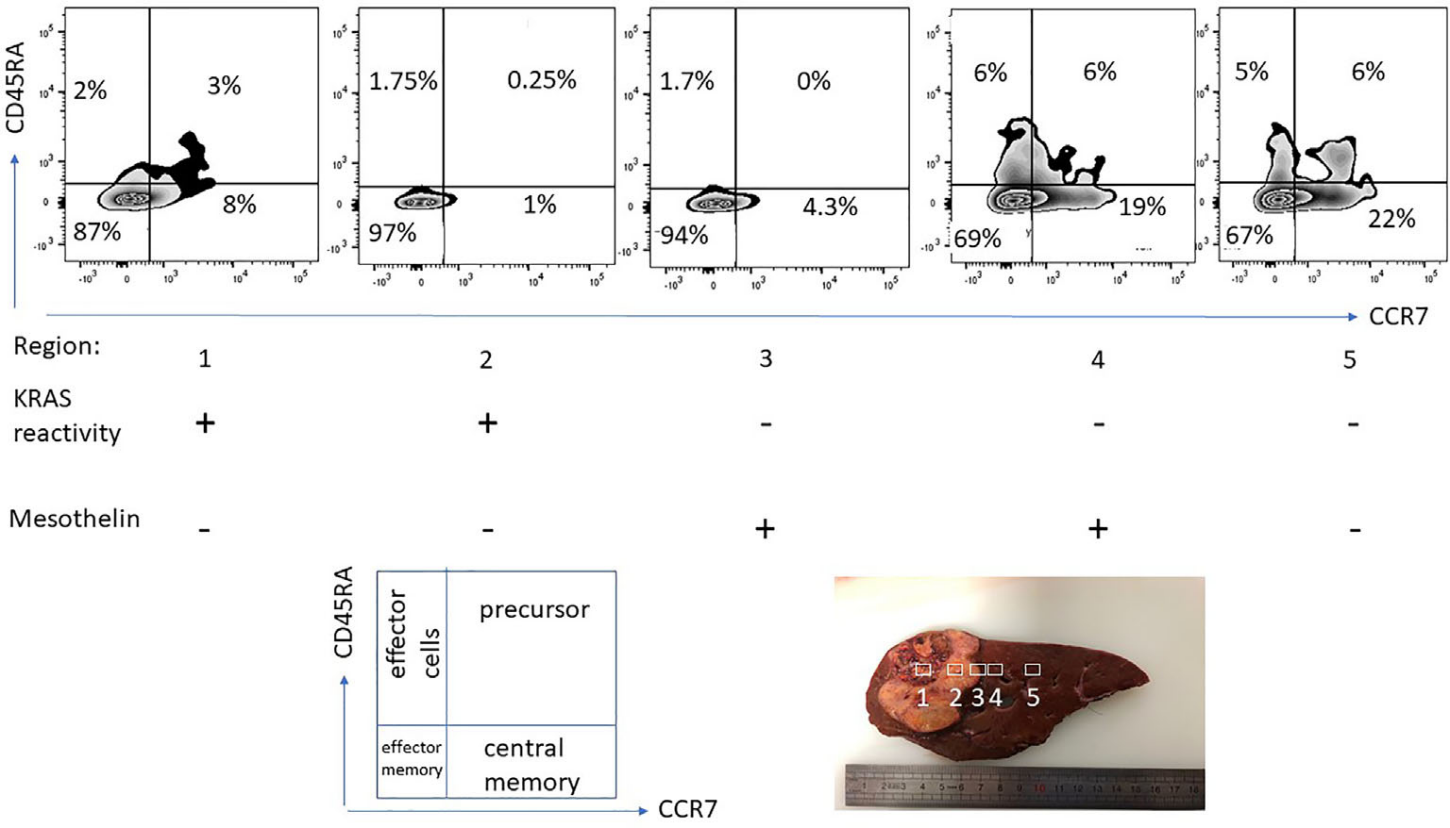
T-cell phenotype and functional-spatial differences. TIL were expanded from different regions from a pancreas cancer lesion metastatic to the liver, 5 regions were harvested in different proximity to the tumor center. Note the different homing/maturation phenotype based on CD45RA/CCR7 expression, central memory T-cells in the tumor periphery. Thus, the quality of the T-cell response (to neoepitopes) is also associated with the immune cell maturation status. Reactivity to (mutant) KRAS or mesothelin was tested by pre-incubation of TIL for 5 days followed by IFN-gamma production analysis. Exclusive KRAS recognition in the tumor center versus mesothelin recognition in the tumor periphery and in macroscopically cancer-negative tissue demonstrating that the selection of neoepitope specific T-cells depends on the anatomical location.

Not only the nature of the tissue specimen, yet also the different anatomical location of the T-cell harvest is critical if T-cells are tested for recognition of neoepitopes, exemplified in **Figure 4**. Not only the frequency of CD4/CD8^+^ T-cells changes in relation from the tumor center to the tumor periphery, also the epitope target specificity changes, mutant KRAS reactive T-cells were detected in the tumor center, anti-mesothelin reactive T-cells were found in the tumor periphery. For clinical usage, it is important to emphasize that the location of the T-cell harvest has to be documented along with caution that different cancer tissue regions harbor different immune cell populations with different neoepitope specificities. While this is not surprising due to the tumor mutanome heterogeneity and consequent TCR diversity, it has to be taken into practical considerations as different areas of cancer specimens are harvested to expand TIL for cellular therapy. While TIL isolation and propagation for immunotherapy is feasible for some cancer types, patients with certain malignancies may present with cancer lesions that are – even with minimal invasive procedures or biopsies – very difficult to access (198). For those cases, PBMCs may be a viable and less invasive option, since peripheral blood T-cells are easily accessible and can be later used as a cell source for T-cell engineering to express a specific TCR or CAR (199, 200). HLA-matched donor-derived T-cells from donor PBMCs reactive to patient-derived neoantigens also present a viable option for neoepitope directed cellular immunotherapy (21).

There is also a different source of T-cells that can be considered for anti-cancer directed cellular therapy and for screening of neoepitope-reactive T-cell population, namely tissue-resident memory T-cells (TRM), a population of non-recirculating CD8^+^ T-cells, residing long-term in peripheral tissues. TRMs contribute to tumor surveillance and to protection against viral and bacterial infections (201, 202), TRMs express a variety of homing markers that allow them to recirculate in peripheral tissues, such as CD103 (αe integrin) and CD49a (collagen-binding molecule antigen-1 (202–204), they produce Th1-type cytokines, namely IFN-γ, tumor necrosis factor alpha (TNF-α) and interleukin 2 (IL-2) upon stimulation (203, 205), yet may also elaborate Th17- or Th2-type cytokines (206–208). The impact of TRM cells in tumor surveillance is also related to the fact that TIL that express TRM cellular markers have been identified in several human solid cancers (209–215) often in correlation with improved clinical outcome (216–220). Therefore, the presence of homing markers and TRM cells among TIL, in addition to the tumor localization from where TIL are being expanded merit more attention in clinical studies pertaining to their role in neoantigen recognition, tumor surveillance and the selection of TIL for improved cellular immunotherapy.

## Conventional CD4^+^ and CD8^+^ T-Cells

T-cells bearing the conventional TCR αβ have been associated with augmenting clinical responses in patients receiving immunotherapy – both cell-based (191) and immune checkpoint inhibitors (221) and, more recently, personalized vaccines (78, 222). Both CD8^+^ and CD4^+^ neoantigen-specific TCR αβ responses in hard-to-treat cancers such as glioblastoma (14, 72), pancreatic malignancies (18), non-small-cell lung cancer (2, 3), melanoma (69, 76), bile duct (40) and colorectal cancers (41) are now regarded as vital to promote durable clinical responses in patients, further to the presence of suitable neoepitope restricting HLA elements (27, 223). Much focus has been placed on CD8^+^ TILs in mediating anti-tumor activity due to their cytotoxic capacity and responsiveness to immune checkpoint blockade in view of their neoantigen-directed immune reactivity (224). In contrast, CD4^+^ T-cells are largely attributed with helper functions, i.e., production of effectors cytokines, such as IFN-γ and TNF-α while responding to stimuli provided by IL-2, IL-6, interleukin 18 (IL-18), and IL-1β to list a few. Nevertheless, the cytotoxic activity of some tumor-directed CD4^+^ T-cell subsets is now considered an important arm of the MHC class II restricted immune defense (174), particularly in patients with a defective HLA-I pathway. Further to autologous TCRs, allogeneic T-cells from HLA-matched healthy donors can either naturally react to – as part of the naturally occurring TCR repertoire – neoepitopes or they can be specifically selected and re-programmed to specifically react to neoepitopes and kill cancer cells without overt off-target toxicity (21, 225). Past and emerging studies consolidate the utility of conventional T-cells as sources of TCRs strongly reactive to peptide-HLA complexes based on the best-fitting epitopes to transduce PBMCs for developing possible ‘off-the-shelf’ TCRs options for patients with cancer expressing distinct tumor-associated antigens and sharing the respective restricting MHC allele (41, 226–230).

## Non-Conventional T-Cells: TCR γδ and Invariant NKT-Cells

The relevance of TCR γδ T-cell subsets has received a substantial deal of attention in the last decade owing to clinically meaningful observations of anti-cancer reactivity in several cancer types (231–236). In patients with malignancies showing a defective HLA system, TCR γδ T-cells may have the upper hand in immune recognition as they do not need the classical antigen presentation machinery for antigen recognition and activation (237). TCR γδ T-cells participate in a wide array of immunological processes which can activate or dampen the ensuing T-cell response, including the production of IL-17 which has been implicated in the pathogenesis of inflammatory disease as well as cancer (238–241). While several different gamma chains are known, two main delta chains have been described in humans TCR γδ T-cells, namely Vδ1 and Vδ2, although Vδ3^+^ T-cells have been isolated from the liver (238, 242). As mentioned earlier, an important feature of TCR γδ cells is their expression of natural cytotoxicity receptors (NCRs), which are also present on NK-cells, namely NKG2D, NKp30 and DNAM-1 (243, 244). The most commonly occurring subclass of these cells are those expressing the Vγ9Vδ2 TCR which, *via* NKG2D, can bind to the HLA class I-like molecules MICA/B, akin to NK cells (244). Daley and colleagues recently showed that γδ T-cells outnumber CD8^+^ T-cells in human pancreatic adenocarcinoma tissues, and potentially dampen the anti-tumor activity of conventional TCR αβ T-cells (245). Although anti-tumor γδ T-cell subsets expressing TCR Vγ9Vδ2 comprise a very small percentage in the tumor microenvironment, approximately 30% of circulating γδ T-cells expressed Vγ9. Apart from these, Vδ1^+^ T-cell subsets have been shown to mediate productive immune responses against gastrointestinal tumors (246, 247), and are likely to be important players – in addition to the much studied Vγ9Vδ2 subset – in developing cellular immunotherapies for cancer (238).

In addition to TCR γδ T-cells, invariant natural killer (iNKT)–cells – bearing the invariant TCR V24α chain may also be relevant in recognizing neoepitopes in cancer (248). Alpha-galactosylceramide-driven activation of iNKT-cells (afore-mentioned CD1d-mediated antigen presentation) in patients with solid tumors has resulted in stable disease and detectable immune responses, including in protocols involving DCs pre-activated with alpha-galactosylceramide prior to infusion into patients (248–250). iNKT-cells can also exhibit cytolytic activity akin to NK-cells and cytotoxic CD8^+^ T-cells, requiring the NCRs NKG2D and NKp44. Considering the characteristics of ‘non-conventional’ T-cells and clinical studies that support their individual use in immunotherapy (251), the combination of these immune cells with αβ T-cells should be considered in order to augment anti-cancer directed T-cell responses.

While isolation and cultivation of autologous T-cells from patients with cancer is a tailor-made drug development strategy, it is also time-consuming and can only cater for a limited number of patients at a time. Importantly, not all patients qualify for surgery and tumor biopsies are not always sufficient for TIL propagation after allocation for histopathological analysis. Therefore, TCRs from peripheral blood T-cells – recognizing shared or common cancer mutations – can be used to generate a cellular product generation with heterologous expression in patient T-cells. This approach has been shown to be successful in a patient with metastatic colorectal cancer who received an HLA-Cw08*02-restricted TCR targeting the KRAS_G12D_ driver mutation (41). An integrated approach using NGS and immunology may be able to identify new neoantigens which are shared among certain patient groups to be adapted for developing transgenic TCR-based cellular drugs. The use of mucosal associated invariant T-cells (MAIT) and their respective targets for the potential use in personalized therapies is not discussed here.

## B-cells

Unlike T-cells, B-cells have received the least attention although emerging evidence suggests that they should be accounted for in future treatment regimens due to their association with beneficial anti-tumor responses, including the production of cancer antigen-specific antibodies (252–254). TIB (tumor infiltrating B-cell) mediated responses, visualized by antibodies recognizing KRAS mutations, have been described in patients with pancreas adenocarcinoma (PDAC) (255), highlighting their clinical utility in anti-tumor immune responses. NGS platforms can supplement innovations in surgical oncology by the use of fluorescently-labelled antibodies and imaging to precisely mark the specific location of cancer disease for resection in the patient (256) coupled with *in vitro* laser microdissection of specific intra-tumoral regions of interest by identifying areas which are likely to represent varying mutational cancer profiles and matching T-cell reactivities (257). New data has revealed that an intact B-cell compartment in patients with advanced melanoma undergoing immune checkpoint blockade therapy (anti-PD-1, anti-CTLA-4 or both) are predictive of improved patient survival, given that no immune-related adverse events (irAEs) occur (258). This observation was associated with an increased proportion of circulating CD21^+^ B-cells and plasmablasts after therapy. Also, the role of antibodies in the recognition of cancer-specific mutated proteins such as KRAS (255) as well as CMV- and EBV-derived epitopes in the TME (259) and carbohydrates (183) cannot be dismissed and warrant deeper insights in clinical studies examining the role of TIBs in immune-recognition and immunomodulation in the tumor. Antibodies binding to neoantigens of interest can be used for designing CARs, provided these neoantigens are expressed on the tumor-cell surface (e.g. MUC4), for which TCRs in blood were recently described (44). Waltari and colleagues recently showed how combining NGS and immunoassays platforms, while incorporating RNA-Seq and downstream bioinformatics analysis followed by *in vitro* stimulation with CpG and clonal expansion, can help identify and isolate memory B-cells from blood with B-cell receptors (BCRs) for a specific antigen in association with protection from disease which, in this case, was influenza (260). A more recent study reported the use of RNA-based Repertoire and Gene Expression by Sequencing (RAGE-Seq) that was able to identify BCR and TCR species circulating in blood of a patient with breast cancer that facilitated the tracking of lymphocyte populations in different tissue compartments (261). Thus, novel innovations in NGS techniques may also aid the discovery of distinct neoantigen- and tumor-reactive lymphocytes with clinical applicability. Neoepitope vaccination strategies may also induce mutation-specific antibodies in antigen-driven expansion of B-cells that may also produce anti-cancer directed cytokines (262), functional TIB (263) that are associated with increased survival (264, 265) have been shown to produce antibodies that target TAAs, including mutant KRAS molecules (255). Thus, neoepitope-based vaccination immuno-monitoring should also include screening of vaccine peptide-specific humoral immune responses in the peripheral circulation as well as in TIL, even if the vaccine peptides are designed for MHC class I or -class II binding.

### Laboratory-Based Platforms to Complement NGS and Facilitate Personalized Immuno-Oncology

A close collaboration with the pathology unit at healthcare facilities and allowing their active involvement in all phases of the clinical trial is crucial. Routine as well as specialized immunohistology panels can be designed to aim at analyzing HLA profiles in patients with cancer before, during and after immunotherapy. Furthermore, antibodies that can differentiate between misfolded and native HLA class I molecules on paraffinized tissue samples would be an immense advantage, since aberrant HLA class I molecules on the surface of tumor cells are likely to be missed by CD8^+^ T-cells. Reagents that recognize all components of the HLA class I pathway such as TAP (151, 266), tapasin, β2-microglobulin-free HLA-A variants (151, 267, 268), LMP2/7 (151) and β2-microglobulin (151) have been described before, while those that recognize HLA class II components are also commercially available. In addition, immunostaining panels for histology, encompassing mutant epitopes of cellular proteins which can identify cancer cells and indicate whether druggable mutations are present in cancer tissue would be of great clinical value. Expression of the TNF-related apoptosis inducing ligand (TRAIL) molecules on the surface of cancer cell may also be a good indicator of their sensitivity to treatment-induced apoptosis (269). This approach may, in fact, serve as a means of ‘companion diagnostics’ to facilitate mutation-directed T-cell therapies. Circulating tumor cells (CTCs) that may be present in liquid biopsies can also be purified for immunocytochemistry (270–272). Two newly published reports describe how stable HLA molecules with an empty epitope-binding groove can be customized to bind peptides of interest and leveraged to screen for the best-fitting epitopes which induce an immune response (273, 274). Indeed, all of these methods could be exploited to screen for best-fitting neoepitopes using information arising from immunohistology and NGS data and obtain a better personalized anti-cancer vaccine and/or another treatment type, that may include pre-incubation of TILs with neoepitopes (to increase the frequency of TIL against mutant peptides). Multiparametric flow cytometry constitutes an integral part of screening for cellular immune responses and their physiological status is an addition to qualifying them for release as cellular products for personalized immunotherapy (55, 275). A wider panel of flow cytometry-compliant antibodies which can assess lymphocyte subsets present in cancer tissue based on phenotype and physiological status (e.g. exhausted vs. active, cytotoxic potential, type of recognition including MHC class I/II, CD1, MIC1A/B, MR1 restricted T-cells) prior to processing for cell culture would be an immense addition to clinical immunotherapy protocols (**Table 1**), augmenting findings from immunohistology analysis of tumor tissue. A dynamic set of flow cytometric analysis panels for further characterization of cellular products over the course of immune cell expansion for adoptive therapies will be of practical help, linked with immunohistology data from the resected tumor specimen. Ideally, functional T-cell data either from *ex vivo* expanded T-cells for adoptive therapy, or T-cell data obtained during immuno-monitoring in the context of peptide-based vaccination will yield extended information which can be amalgamated to the IHC data. Recent studies show that TP53 and KRAS mutations increase the expression of PD-L1 on tumor cells (276, 277), indicating that the presence of shared mutations can also be used as a companion diagnostic readout in personalized immunotherapy protocols.

**Table 1:** Lymphocyte markers for use in IHC and flow cytometry studies to support clinical decision making in personalized cancer immunotherapy.

Lymphocytes	Standard Analysis	Additional	Remarks
T-cells (TCR αβ, TCR γδ, NKT, MAIT cells)	CD3, CD4, CD8, CD25, TCR Vα/Vβ, TCR Vγ/Vδ, CD56, classical MAIT TCR Vα 7.2	NKG2D	Cytotoxic effector molecule (also applies to NK-cells)
		PD-1	Immune checkpoint molecules
		CTLA-4	
		LAG-3	
		TIM-3	
		IL-7R	IL-7 receptor/CD127; for Treg identification
		4-1BB	CD137; activation marker
		CD45RA	To assess the memory phenotype of T-cells
		CCR7	
		CXCR3	To assess the T-helper phenotype and tissue-penetration capacity of T-cells
		CCR4	
		CCR6	
		FoxP3	Transcription factor upregulated in activated T-cells and Tregs
		Helios	Aids in Treg identification
		Perforin	Cytolytic effector molecule
		Granzyme, Granylysin	Apoptosis-inducing effector molecule
		CD8+CD69-CD39-	CD8+ TIL with stem cell like properties and a CD69/CD39- phenotype are associated with response to therapy
		Cytokine receptors i.e., IL-6R, IL-1βR, IL-18R, IL-21R	For T-cell activation by APCs, and may help identify high-affinity antigen-specific cells
		BTN3A1/CD277	Antigen presentation to γδ T-cells
		IL-17	Can be useful as a marker for potentially pathogenic γδ T-cells
		Fas	Involved in apoptosis induction
		FasL	
B-cells (also act as APCs)	CD19, CD20	CD21	May have positive prognosis for patients with cancer
		FasL	Involved in apoptosis induction
		Fas	
		HLA class I pathway components	HLA alleles, TAP, tapasin, LMP2/7, β2M; to predict response to immunotherapy
		HLA class II pathway components	HLA-DR/DMA/DMB/DOA/DOB; to predict response to immunotherapy
		BTK	Bruton tyrosine kinase; may impede anti-tumor responses

## Peptide-HLA Stability Assessment

In addition to predicted HLA-matched neoepitopes, the use of an effective *in vitro* assay to measure the strength of peptide binding and stability may be able to improve the decision-making in selecting the most suitable neoantigens for personalized vaccine design. The measurement of the stability and half-life dynamics (also referred to as the ‘off-rate’), which informs of how long a given peptide sequence can bind to the groove of the HLA molecule, was previously shown in the context of the TAA survivin (278), *Mycobacterium tuberculosis* protein TB10.4 (279, 280), HIV-1 epitopes (281), HA-1His autoantigen (282) and recently using HLA-B07*02-restricted myeloperoxidase (MPO) epitopes (226), allowing for the selection of strong binders capable of inducing optimal T-cell recognition and cytokine responses and/or cytotoxicity (283, 284). The half-life of the peptide-HLA complex class I/II constitutes an important parameter in dictating immunogenicity – the duration of time for which the peptide-HLA complex can be stably be expressed on the surface of the APC (including transformed cells) and evoke a strong CD4^+^ or CD8^+^ T-cell response (281, 283, 285–288). A recent study demonstrated that the stability of the peptide appears to be a better correlate of immunogenicity than affinity (287), and this may suggest that highly stable epitopes (those with a long half-life) can have a very strong affinity for their cognate HLA allele (283, 284, 289). The evaluation of peptide recognition defined by IFN-*γ* production – as a result from tumor mutanome analysis either in the format of synthetic peptides or as minigenes – can be used to gauge for biological activity in TIL as proficiency assay to gauge for T-cell reactivity against commonly strongly expressed mutant or non-mutant tumor associated antigens (290).

The absence of IFN-γ in the TIL culture supernatants from assays probing antigen reactivity does not necessarily imply that the predicted peptides do not induce any type of effector response by the candidate testing T-cells. For instance, matching TCR-HLA immune synapses may also lead to cytotoxicity instead of cytokine production, which can be determined by measuring surface CD107a induction or Fas expression. Production of granulocyte-macrophage colony-stimulating factor (GM-CSF) in lieu of cytotoxicity by CD8^+^ T-cells has been shown in the context of HLA-A1-restricted melanoma epitopes (291). The absence of cytokine production may also stem from the fact that the predicted neoepitope is not naturally processed and presented to the immune system. Alternatively, some of the TCRs which may recognize predicted peptides are present in the general TCR repertoire of the patient but they may not be present in the tissue sample harvested to test for T-cell recognition of the predicted epitopes. For instance, TIL may represent a rather focused and antigen-specific enriched TCR repertoire (29, 292) and PBMCs from a standard blood draw represent usually 2% of the entire T-cell pool. One of the ‘gold’ standards in gauging anti-neoepitope-specific T-cell responses is certainly whether peptide-reactive T-cells – after sorting by tetramers, IFN-γ–capture or by using activation markers (e.g. CD137)-are able to recognize the patient’s own tumor cells. This would strongly argue that epitope-specific T-cells recognize as well naturally processed and presented peptides – and that the selected candidate epitopes are biologically relevant. A different ‘reversed’ procedure is the repetitive stimulation with autologous tumor cells and the subsequently enriched T-cells are then tested for epitope specific reactivity (18).

## ‘Real World’ Data: Approach at the Champalimaud Clinical Centre

The points discussed above guide the immunotherapy program at the Champalimaud Foundation termed ‘ImmunoSurgery’ to underline i) the seamless connection with the surgical team and the subsequent examination of the resected tissue specimens from a clinical pathologist. The tissue used to produce TIL – and to perform tumor exome sequencing – requires documentation and histopathological information about the functional loco-regional diversity of the T-cell infiltrate into the tumor tissue which can be further assessed by deep TCR sequencing, ii) that T-cells expanded from surgical specimens are tested for neoepitope specificities as defined by tumor mutanome analysis and represent a ‘biological knife’.

**Figure 5:**
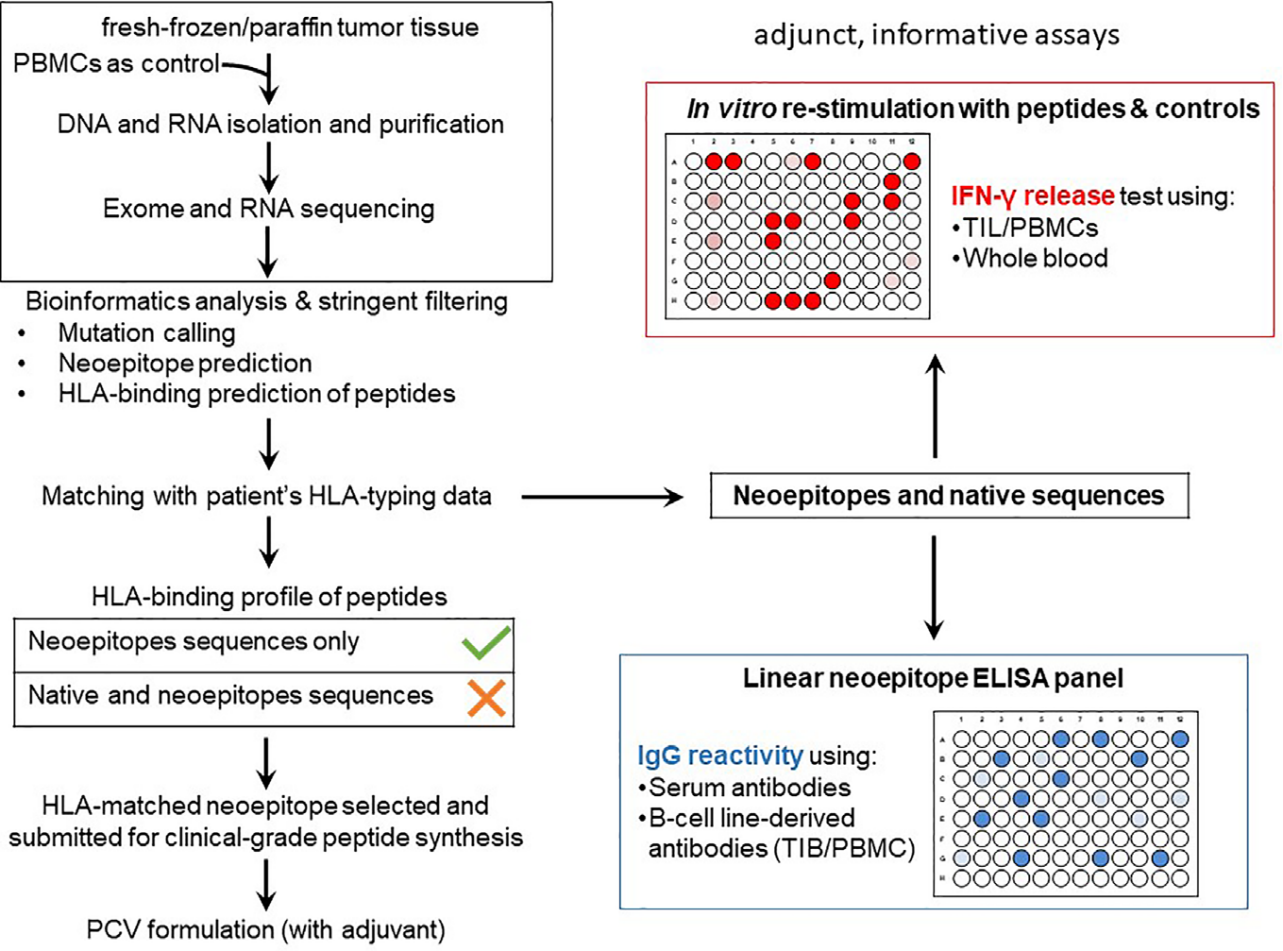
PCV development and immuno-analyses workflow at the ImmunoSurgery Unit. Formalin-fixed paraffin-embedded (FFPE) or fresh-frozen tissue samples prepared by the Pathology Unit at the CCC is submitted for WES of tumor DNA with the patient’s PBMCs as an internal control for downstream analysis, RNA-Seq is also sometimes performed to tumor RNA. The WES and RNA-Seq raw data is then analyzed at the ImmunoSurgery Unit at the CCU to predict private mutations followed by HLA class I and II binding prediction matched to the patients’ HLA restriction profile to select candidates for inclusion in the PCV formulation. Only HLA-binding, neoepitope-containing peptides but not the wildtype counterparts are considered. The same and also 15-mer equivalent but non-clinical grade peptides, alongside the corresponding native sequences, are used for gauging TIL and/or PBMCs reactivities based on IFN-γ production (the peptide is at a concentration 1ug/mL tested with 10e^4^ responder T-cells; the fixed T-cell number allows to compare results obtained at different timepoints or from tissues harvested from different tumor areas). This part of the immunological evaluation of the neoepitopes is used in the follow-up phase of the trial which aims to assess T-cell responses of patients to the PCV and TIL therapies (possibly also for patients receiving immune checkpoint inhibitors). A different platform is an ELISA panel comprising the patient’s neoepitopes in linear format to assess IgG reactivity using antibodies from serum as well as those secreted by TIB and PBMC-derived B-cell lines. Neoepitopes are also screened for TIL recognition since TIL are routinely generated to gauge for differences in TIL versus PBMC recognition. This will allow to describe whether selected neoepitopes are recognized in the tumor lesion that was used to identify the tumor neoepitopes (by NGS), it also allows to screen for differences in TIL recognition from tumor lesions harvested at different anatomical sites or at different timepoints in the course of the disease.

**Figure 6:**
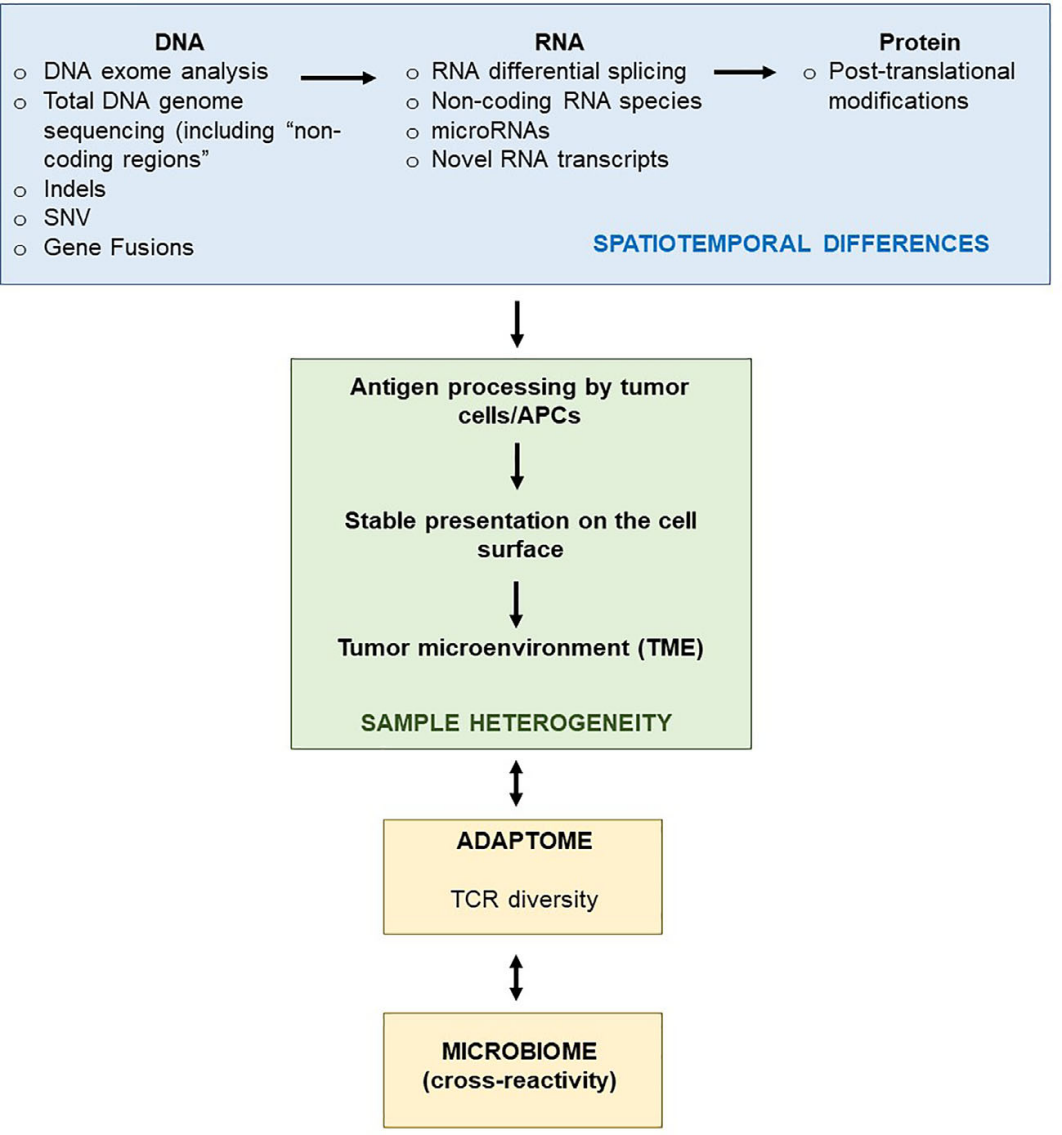
Schematic representation of the general molecular paradigm of neoantigen recognition in the TME. The process of transcription of DNA to RNA and then to protein (antigens) is prone to generate heterogeneity in the context of cancer, i.e., the same DNA molecule may be differentially transcribed (due to RNA alternative splicing or mutations) and then translated to different proteins isotype (also as a result of post-translation modifications) or there might be gene fusions that result in novel RNA transcripts. The heterogeneous expression of tumor antigens, as a result of spatial-temporal differences in DNA to antigen production, results in different antigens being presented to the immune system by HLA complexes (as well as whole antigens) at the cell surface of a tumor cell and, therefore, contributing to different sub-regional TMEs within the same tumor tissue sample. These are likely to be neoantigens, as they are not present in healthy (non-transformed) tissue. The TCR diversity (“adaptome”) will also change depending on the specific TME, i.e., different TCRs will be encountered depending on intratumoral spatial differences. Along the same lines, molecular structures associated with the microbiome present in the tumor tissue may cross-react with some T-cells, depending on the presence of absence of TCRs that recognize such microorganisms. The possible cross-reactivity, if present, may favor the expansion of the relevant immune-cell populations and, therefore, change the TCR repertoire.

**Figure 7:**
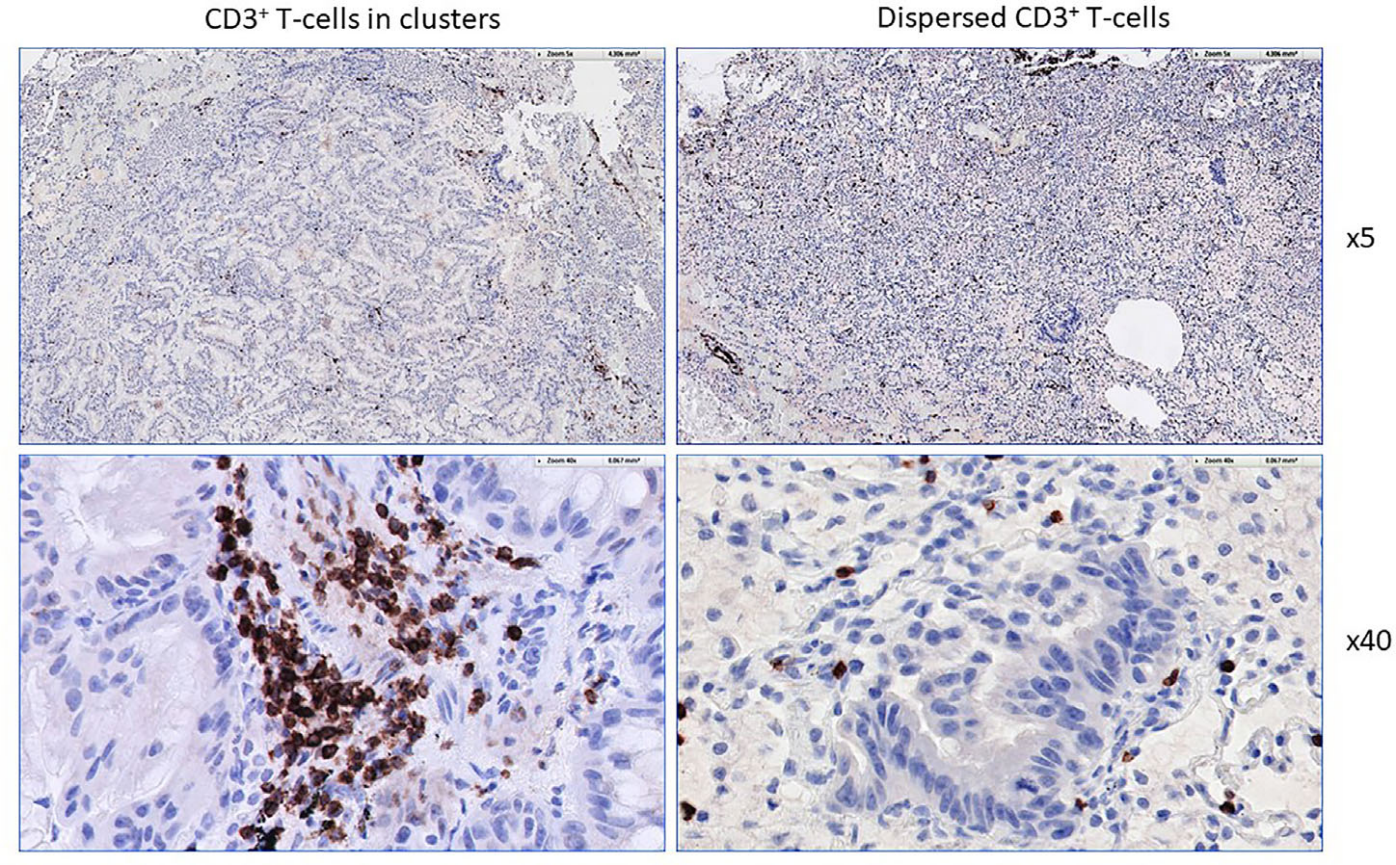
Different immune-textures in cancer lesions. Starting point for WES and RNA-Seq. Definition and documentation of the immune cell infiltrate. Parallel slices of the paraffin-embedded tissues are procured and subjected to DNA and RNA analysis. Note the different patterns of CD3^+^ T-cell clusters (left) versus individual CD3^+^ T-cells in close proximity to tumor cells. RNA isolated from this tumor section would also allow for deep TCR-sequencing and allow to trace back individual TCR CDR3 motifs in case if neoepitope specific TCRs are identified.

**Figure 8:**
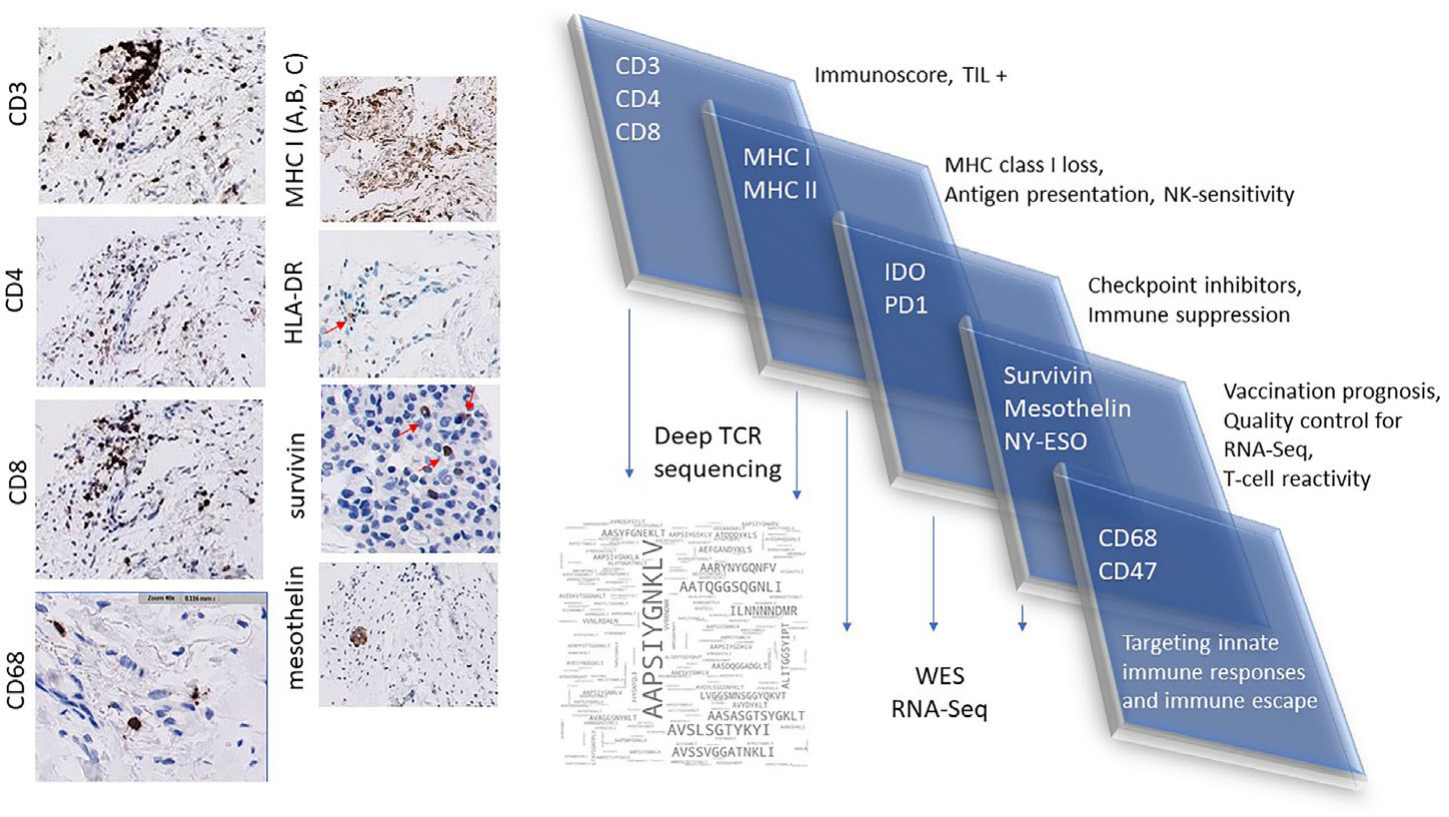
Example of a standard immuno-histological analysis of a tumor sample at the Clinical Pathology Unit. Analysis of CD3^+^, CD4^+^ and CD8^+^ T-cell infiltrates along with tumor-associated CD68^+^ macrophages. Testing for MHC class I (HLA-A, B and C) expression to screen whether transformed calls can be recognized by CD8^+^ T-cells, general MHC class I loss would not support vaccination strategies of adoptive T-cell therapy targeting TCR alpha/beta T-cells as the immune effector population. CD47, PD-1 and PDL-1 expression to gauge immune escape. Examination of commonly shared, non-mutant TAAs (NY-ESO-1, survivin, mesothelin) to identify T-cell responses in TIL and in corresponding PBMCs. Expression analysis of TAAs aids in quality control concerning RNA-Seq (of the corresponding gene coding for the TAA) and deep TCR analysis of T-cells reacting to TAAs.

The workflow used for tissue procurement, neoepitope identification and T-cell screening is provided in **Figure 5**, factors that may impact on the nature of neoepitopes, neoepitope generation and factors shaping the responding T-cell repertoire are compiled in **Figure 6**. Tumor-epitope identification by WES and RNA-Seq is guided by careful selection of the tumor area for genetic analysis. A more recent excellent review addressed the clinical utility of neoantigen identification, peptide processing and MHC presentation of candidate epitope targets for rational vaccine design (119). Ideally, a tumor area that shows more than 80% of transformed cells is selected (**Figure 7**). In order to better define the tumor specimen, a standard analysis for the immune-contexture is carried out at our institution. A general (HLA-A, B and C) MHC class I loss would exclude patients from entering into peptide vaccination trials. CD3^+^, CD4^+^ and CD8^+^ T-cells are being described along with the presence of CD68^+^ macrophages, the presence of MHC class I antigens (HLA-A, B and C), the presence of HLA-DR, the expression of tumor-associated antigens (e.g. NY-ESO-1, mesothelin or survivin) to test for T-cell responses directed against non-mutant target antigens, as well as molecules associated with immune-suppression/evasion (e.g. PD-1, PDL-1 and CD47) along with the description if immune cells reside within the tumor or rather around, as single cells, or in clusters (**Figure 8**). The immune microenvironment imposes a strong pressure in untreated non-small-cell-lung cancers that subsequently show different routes of immune evasion. Different qualities of immune cell infiltration are associated with immune-editing (and therefore the diversity of neoepitopes available for T-cell expansion), MHC loss and defects in the antigen processing and presentation pathways (293). This may be differently associated with distinct tumor locations, ideally, parallel immunohistological sections are selected for WES and RNA-Seq. We combine different platforms to identify mutations in tumor exome data, combining the results of four different tools [Mutect2 (294), Varscan2 (295), Strelka2 (296) and Lancet (297)] and keep only mutations that are identified by at least two of these platforms. pVACtools takes results from the exome sequencing, complemented with mutations and fusions found in the transcriptomics data set which is then combined with several prediction algorithms resulting in a consensus ranking of neoantigens based on four criteria: rank of peptide binding affinity to the nominal MHC allele, rank of fold change between mutant and wild-type alleles, rank of mutant allele expression and the rank of DNA variant allele fraction (298). We test routinely two peptide formats to screen for cancer associated antigens in TIL or in PBMCs, namely i) peptides with 15 residues where the mutation is centered (and 7 amino flanking the mutation) or ii) the full downstream protein sequence in case of a frameshift. These different formats are used for immunoassays to gauge for INF-*γ* production in a 96-well format and supernatants are harvested at days 3 and 7 (see **Table 2** for references). Peptides of different lengths tailored according to the MHC typing of the patient are selected for candidates for PCVs based on i) if they are driver mutations, ii) strong expression in RNAseq, iii) superior binding of the mutant epitope as compared to the wildtype sequence, iv) frameshift mutations and v) practical considerations concerning peptide synthesis. If there are obvious different areas in the tumor specimen (**Figure 7**), micro-dissection of such areas is considered to estimate differences in tumor-heterogeneity. We are currently testing whether neoepitope directed T-cell responses are different in the primary cancer lesion as compared to metastatic lesions – that are usually harvested at a later time point during the cancer disease. Recognition analysis of MHC class I or -class II restricted epitopes defined by IFN-*γ* production in PBMCs versus TIL as a predictor of which neoepitopes are most likely immunogenic and also lead to clinically relevant responses in the course of a peptide-based vaccination strategy can only be tested in a phase I clinical trial that is currently being prepared.

**Table 2:** Examples of molecular analysis guiding future therapeutic decision making.

Analysis	Examples of target genes	Potential biological and clinical effects	Potential practical consequences	Reference
**WES or RNAseq** Mutations in immunological response genes, i) e.g. induced by radiation, ii) germline mutations, iii) or natural variants that impact on immune function.	Immune responses genes in innate or adaptive immune responses including immune cell signaling, e.g. C2, CD163L1, FCγR2A	Gene variants or mutated genes edit immune infiltration, quality and quantity of the tumor-microenvironment	Despite identification of neoepitopes for neoepitope vaccination therapy plus checkpoint inhibitors, the innate or adaptive immune response may be blunted. The anti-cancer vaccination effect may not be achieved due to the incapacity to mount strong and cancer antigen specific immune response. Other therapeutic strategies are to be considered	(299, 300)
**CHIP analysis, WES and RNAseq**	Not only mutations in *bona fide* immune response genes, yet in cancer-associated genes, i.e. ARIDA, shape the quality of immune responses and T-cell infiltration	ARID1A aberrations may lead to differential chromatin accessibility and therefore to blunted anti-cancer directed immune responses, e.g. by reduction of overall IFN-gamma production, diminished immune cell infiltration and insufficient long- term immune memory responses.	Awareness that immunological treatment strategies may be challenging due to reduced IFN-gamma production. Detailed molecular analysis may aid to decipher how an effective anti-cancer directed milieu could be achieved without ARIDA1A interference	(301)
**Deep TCR sequencing**, TCRalpha, beta, gamma and delta chain. Bulk sequencing may be sufficient for most clinical questions. Single cell analysis possible.	Detailed molecular description of TCR infiltrate to objectively describe the situation prior to therapy. Different TCR repertoires in spatiotemporal cancer lesions.	A focused TCR repertoire can represent a relevant clonal immune response. Clonal immuno-editing may occur and lead to antigen – loss variants. ‘Clonal replacement’ appears to be associated with response to checkpoint inhibitors.	TCR convergence in PBMCs or tumor lesion (biopsies) and/or clonal convergence as companion diagnostics for immunological treatments. Knowledge of neoepitope specific TCR allows to follow antigen-specific reactivities. Broader TCR repertoire may provide more possibilities to react to neoepitopes imposed by the structural constraints of the MHC – peptide complexes. Long term neoepitope specific responses have been identified in patients with melanoma after peptide vaccination with different TCR clonotypes (directed against the identical epitope, this allows to link epitope-specific recognition with TCR diversity and functional avidity.	(116, 302, 303)
**Epitope specific recognition** defined by IFN or other cytokines in TIL from surgically removed tumor specimens and PBMCs	Either ‘private mutations’ or commonly shared tumor – associated antigens, i.e. NY-ESO-1, mesothelin, or common infectious pathogen antigens, e.g. EBV or CMV, provide a ‘recognition fingerprint’ to follow the immune response pattern in immunological therapies	Standard chemotherapy or immunological therapies shape the immune-competence to indicator targets (private antigens, TAAs or infectious disease targets).	Loss of anti-EBV or CMV recognition in peripheral blood, or anti-tumor antigen directed T-cell responses may represent one factor in the complex decision making choosing second or third line treatment therapies.	(259, 304–306)
**Immuno-histology, RNA expression** of commonly shared tumor associated antigens	NY-ESO-1, survivin or mesothelin expression	Commonly shared TAA-vaccines, e.g. anti-survivin, mesothelin or NY-ESO-1 are available. Anti-Mesothelin CARs or transgenic TCRs. MHC class I or class II-restricted NY-ESO-1 restricted TCRs are in clinical trials.	Strong antigenic heterogeneity in solid tumors defined by neoepitopes may still allow to use the immunogenic cancer – testis antigen NY-ESO-1 if sufficiently expressed. Mesothelin CARs have shown to be associated with epitope spreading and induce T-cell responses against private antigens. Commonly shared TAAs may represent a cellular ‘first line’ treatment, enhancement possible with checkpoint inhibitors.	(307–310)
**WES and RNAseq** bulk or single sequencing	Clonal spatiotemporal evolution in metastatic cancer lesions	‘Immuno-edited’ tumor clones may be eliminated during the course of the tumor disease while progressing tumor clones are ‘Immune-privileged’ despite the presence of tumor-infiltrating lymphocytes. Neoantigen depletion was observed in tumors with high Immunoscore and spatial proximity between tumor cells and T-cells.	‘Immuno-edited’ tumor lesions may still be accessible to commonly shared TAAs. If neoepitope-directed therapies are contemplated, a ‘fresh’ tumor biopsy after chemotherapy or immunotherapy is advisable due to the tumor evolution in order to obtain the most ‘updated’ antigenic profile.	(311)
**WES in tumor versus metastasis**	Mutanome in association with spatiotemporal differences.	Standard chemotherapy or immunotherapy may drive private mutations and clonal evolution: Treated metastases exhibit private ‘driver’ mutations more frequently as compared to untreated metastases.	Private mutations bear the risk of chemoresistance. Obtain clinical material from the most recent cancer lesions to assess spatiotemporal differences of mutations in case if ‘druggable’ targets are considered or neoepitope-directed therapies.	(312)
**Immunological landscape analysis,** RNAseq and/or immuno-histology	Cytokines, such as TGFbeta or IL-17.	TGFbeta may be strongly immuno-suppressive, promote desmoplastic changes in the TME that further inhibit anti-cancer immune responses, IL-17 may drive tumorigenesis.	A strong immuno-suppressive TME may counteract anti-cancer directed immunotherapies, e.g. neo-epitope-directed vaccination. Anti-TGFbeta directed therapies could be considered, either in the format of monoclonal antibodies or – in the case of active cellular therapies, gene-edited (TGFbeta-receptor) negative T-cells.	(313)

In general, the resected cancer specimen is the result of already immuno-edited cancer cell clones, areas of potential neoantigen depletion and clinical tumor progress, despite the presence of immune infiltrates (24). The timing of cancer lesion harvest is also clinically relevant in the context for vaccination, if we presume that the landscape of tumor mutations within the same tumor lesion, and also at different spatiotemporal lesions, represents an active process between cancer evolution and the immune system. Not only tumor cells may be edited, also the available T-cell repertoire undergoes selective pressure. Timing of the tumor lesions for selection of vaccine epitopes determines the mutational burden, yet also the TCR repertoire that changes over time (314). These very basic considerations bear very concrete consequences, i.e., usually tumor specimens are collected to choose mutant target epitopes in vaccine trials should be harvested for analysis after the most recent (standard) chemotherapy or immuno-therapy to reflect potential changes in the neoantigen landscape. Some of these practical considerations that are already currently discussed in clinical decision making or considered in future clinical trials are listed in **Table 2** where we also list the impact of tumor mutations or mutations in immune response genes on anti-cancer directed immune responses. The list of immune response genes that are particularly scrutinized and reported in the course of a standard WES is listed in the **Supplementary Data Set 1**. Although a high mutational burden is generally viewed as beneficial to elicit clinically relevant tumor responses, this may be less accentuated if the tumor lesion is very heterogenous (3), also reflected in the ‘hot’ and ‘cold’ areas in the same tumor specimen (293). Also, a ‘low’ mutational burden does not necessarily imply that neoepitopes within a tumor lesions are not able to elicit biologically and clinically relevant T-cell responses, as evidenced by glioblastoma-specific T-cell expansion, discussed above, and that clonally expanded T-cells, even in ‘low-mutational burden’ tumors (e.g. rhabdoid tumors) show tumor-specific T-cell responses (315). The concordant analysis of the tumor-associated neoepitope landscape does not only allow to link immuno-histological detection of T-cell responses with mutational events, it also enables the spatiotemporal analysis of the molecular composition of the T-cell repertoire with tumor mutations. The TCR landscape, defined by deep TCR sequencing allows the identification of motifs in TCRs infiltrating into tumor tissue as compared to non-transformed tissue (292). A more detailed discussion of this topic is not subject of this review. Yet it may allow to validate – although most likely not at this point in a routine fashion – whether MHC-peptide specific clonal TCRs are present within cancer or tissue lesions by modeling T-cell MHC epitope specificity (316) using yeast-display libraries of MHC-peptide complexes tested for TCR recognition as shown for TIL in colorectal adenocarcinoma (317). This has also been confirmed for the identification of pathogen-specific epitopes – starting with T-cell receptor sequences (318). Thus, *ex vivo* identification of mutant epitope targets may be validated by the identification of the nominal TCR ligands modeling the TCR and MHC-peptide interaction which is beyond the scope of this review.

## Conclusions

Prediction of the best neoepitope candidates for immunotherapy is a multistep process combining several technology platforms ranging from NGS to histopathology and cellular assays. The specifics of the predicted neoepitopes, e.g. defined by the interaction with the nominal classical or non-classical MHC molecules, play a central role in developing clinical products in designing PCVs or in gauging TIL reactivities in association with the spatiotemporal diversity. New findings from translational and clinical research efforts would need to account for different genetic backgrounds and TCR diversities in order to objectively describe differences in immune cells capable of reacting to tumor-associated antigens with the goal to advance personalized cancer immunotherapy to expand potential treatment modalities for patients with cancer.

## Author Contributions

EdS co-wrote the first draft and created figures. JL performed experiments and edited text. AB performed immunohistology. GP performed experiments. CCon performed experiments and wrote passages concerning T-cell subsets. JK and PA contributed to peptide vaccine overviews and edited. NF provided surgical specimens. CCar edited and conceptualized. MC-M edited and performed immunohistology. ZW edited and commented on TCR analysis. DL edited and commented on the HLA and NK part. MR wrote the first draft and combined the overall efforts for the review. MM initiated the work, wrote the final draft, and conceptualized the entire program concerning epitope-based vaccination. All authors contributed to the article and approved the submitted version.

## Funding

This work has been funded by the Champalimaud Foundation.

## Supplementary Material

The Supplementary Material for this article can be found online at: https://www.frontiersin.org/articles/10.3389/fimmu.2021.592031/full#supplementary-material

## Conflict of Interest

The authors declare that the research was conducted in the absence of any commercial or financial relationships that could be construed as a potential conflict of interest.
