# Supplementary material for: Targeting Neoepitopes to Treat Solid Malignancies: Immunosurgery
Source: Front Immunol. 2021 Jul 15;12:592031. doi: 10.3389/fimmu.2021.592031 (PMC8320363; doi:10.3389/fimmu.2021.592031)
Supplement: Supplementary file 1 [file DataSheet_1.pdf]

# Mutation Analysis
# Whole Exome Sequencing (WES)

Champalimaud
Foundation

**Supplementary Data Set 1: List of immune response genes or tumor – associated genes that are particularly scrutinized in the course of tumor WES.**

***List of genes associated with immune – surveillance and DNA repair** (CF-Version0.3.March.25.2018)*

| Gene | Description |
|---|---|
| ***Canonical MHC class I pathway*** | |
| HLA-A | Major human HLA class I complex for presenting protein antigens |
| HLA-B | Major human HLA class I complex for presenting protein antigens |
| HLA-C | Major human HLA class I complex for presenting protein antigens |
| B2M/IMD43 | Beta-2-microglobulin; integral part of MHC-I complex |
| TAP1 | Transporter associated with antigen processing 1; export of nascent peptides into the ER for MHC-I loading |
| TAP2 | Transporter associated with antigen processing 2; export of nascent peptides into the ER for MHC-I loading |
| CALR | Calreticulin, involved in antigen processing and loading |
| PSMB5 | Proteasome subunit beta type-7; belongs to catalytic unit; important for generating peptide antigens |
| PSMB6 | Proteasome subunit beta type-6; belongs to catalytic unit; important for generating peptide antigens |
| PSMB7 | Proteasome subunit beta type-7; belongs to catalytic unit; important for generating peptide antigens |
| LMP7/PSMB8 | Proteasome subunit beta type-8, belongs to catalytic unit; important for generating peptide antigens |
| TAPBP | Tapasin, involved in the interaction between MHC class I and TAP for peptide loading |
| LMP2/PSMB9 | Proteasome subunit beta type-9, belongs to catalytic unit; important for generating peptide antigens |
| MECL1/PSMB10 | Proteasome subunit beta type-10; belongs to catalytic unit; important for generating peptide antigens |
| PDIA3/GRP58 | Protein disulfide-isomerase A3/glucose-regulated protein, 58-kD; involved in export of fully formed MHC-I-peptide complex from ER |
| CNX | Calnexin, involved in folding of MHC-I molecules in ER |
| ***Canonical MHC class II pathway*** | |
| HLA-DR | Human Leukocyte Antigen – antigen D Related (DRA, DRB1,3,4,5) |
| HLA-DQ | Human Leukocyte Antigen, class II, implicated in autoimmune pathology (DQA1 & DQB1) |
| HLA-DP | Human Leukocyte Antigen, class II, implicated in GVHD (DPA1 & DPB1) |
| HLA-DOA | HLA class II histocompatibility antigen, DO alpha chain; forms dimer with DOB |
| HLA-DOB | HLA class II histocompatibility antigen, DO beta chain; forms dimer with DOA |
| HLA-DMA | HLA class II histocompatibility antigen, DM alpha chain; forms dimer with DMB; involved in MHC-II peptide loading |
| HLA-DMB | HLA class II histocompatibility antigen, DM beta chain; forms dimer with DMA; involved in MHC-II peptide loading |

| | |
|---|---|
| CD74 | HLA-DR invariant chain; can also be epxressed on the cell surface; involved in formation and transport of HLA-DR |
| CLIP | Class II-associated invariant chain peptide; keeps MHC-II peptide-binding groove from binding of self-peptides until start of interaction with HLA-DM |
| CaM | Calmodulin; Ca2+ chaperone; probably involved in the generation of MHC-II antigens in cells |
| RAB5 | GTPase involved in phagolysosome formation, which is essential for the generation of antigenic peptides that bind to the MHC-II groove |
| CIITA | MHC-II transactivator; important for IFN-γ-driven expression of HLA-DR |
| ***Non-canonical MHC class I molecules*** | |
| HLA-E | HLA class I histocompatibility antigen, alpha chain E; modulate NK-cell cytotoxicity |
| HLA-G | HLA-G histocompatibility antigen, class I, G; has a role in immune tolerance and also prevents NK-cell cytotoxicity |
| MICA | MHC class I polypeptide-related sequence A; ligand for NKG2D on gamma-delta (γδ) T cells, NK cells and conventional CD8 T cells |
| MICB | MHC class I polypeptide-related sequence B; ligand for NKG2D on gamma-delta (γδ) T cells, NK cells and conventional CD8 T cells |
| CD1a | Presentation of lipid antigens to T cells; present on professional APCs |
| CD1b | Presentation of lipid antigens to T cells; present on professional APCs |
| CD1c | Presentation of lipid antigens to T cells; present on professional APCs |
| CD1d | Presentation of lipid antigens to NKT cells;present on professioal APCs and other cell types |
| CD1e | Intermediate form of CD1 molecule that is expressed intracelularly |
| ***Surface molecules associated with T-cell function*** | |
| CD2 | Also known as lymphocyte function-associated antigen-2 (LFA-2); expressed on T cells and NK cells; requires for migration; might also play a role in activation by co-stimulation during immune synapse |
| CD3 | Indispensable co-receptor which not only identifies T cells but also is pivotal in engaging the peptide-MHC complex to trigger T-cell activation. Also important are immunoreceptor tyrosine-based activating motifs (ITAMs) found in the cytoplasmic tail of CD3 (zeta chain, CD247), contains ITAMs necessary for ZAP-70 binding and downstream signalling to initiate T-cell responses |
| CD4 | Important helper T cell recognition receptor; attaches to MHC-II during immue synapse; also binds IL-16 |
| CD6 | Expressed on T-cell surface and has a role during the immune activation phase |
| CD7 | Expressed on mature T-cell surface and has a role in T-cell activation and engagement with B cells |
| CD8A | CD8 alpha chain; Important surface receptor to identify cytotoxic T cells; binds to MHC-I during immune synapse |

| | |
|---|---|
| TRA/IMD7 | T-cell receptor (TCR) alpha locus; required for TCR structure formation |
| TRB | TCR beta locus; required for TCR structure formaton |
| TRAJ10 | TCR alpha joining 10; required also for generation of TCR repertoire diversity |
| CD80/B7-1 | Expressed on target cells and APCs; binds to CD28 to induce T-cell activation or CTLA-4 to induce tolerance |
| CD86/B7-2 | Expressed on target cells and APCs; binds to CD28 to induce T-cell activation or CTLA-4 to induce tolerance |
| CD28 | Expressed on T cells; involved in T-cell activation by binding to CD80/86 |
| BTN | Butyrophilins; members of this large famiy of B7-related surface-bound molecules are involved in immune cell signalling, largely focussed on T-cell activation and function |
| BTN3A1 | Butyrophilin subfamily 3 member A1; activation of Vγ9Vδ2 T cells |
| PDCD1 | Programmed cell death 1 (PD-1); mainly expressed on T cells; some tumour cells too; inhibits T-cell function |
| PDCD1LG1/CD274 | PD-1 ligand 1; expressed on target cells, including cancer cells; impedes T-cell function |
| PDCD1LG2/CD273 | PD-1 ligand 2; expressed on target cells, including cancer cells; impedes T-cell function |
| CTLA4/CD152 | Cytotoxic T-lymphocyte associated antigen 4; mainly expressed on T cells; binds to CD80/86; inhibits T-cell function |
| HAVCR2/TIM3 | T-cell immunoglobulin and mucin-domain containing-3; mainly expressed on CD8 T cells; activated by galectin-9; inhibits T-cell function |
| LAG3/CD223 | Lymphocyte-activation gene 3; mainly expressed on T cells; binds to MHC-II molecules; inhibits T-cell function |
| VISTA | V-domain Ig suppressor of T cell activation; mainly expressed on myeloid cells; inhibits T-cell function |
| FAS/CD95 | first apoptosis signal (Fas) receptor; expressed on a variety cell of types; can be overexpressed on tumour cells; binds to FasL on T cells to block apoptosis and allow immune evasion |
| FASLG/CD95L | Fas ligand; bind to FasR; expressed on T cells |
| NKG2D/KLRK1 | Also known as CD314; expressed on CD8 T cells, NK cell, γδ T cells; involved in cytotoxic activity upon recognition of and binding to non-canonical MHC-I molecules i.e. MICA/B on target cells |
| OX40 | Alos known as tumor necrosis factor receptor superfamily, member 4 (TNFRSF4), CD134 or OX40 receptor; expressed on activated T cells |
| OX40L | OX40 ligand, also known as CD252; expressed of dendritic cells as well as tumour cells; binds to OX40R/CD134 |
| CD62L/SELL | Also known as L-selectin; binds to GlyCAM-1; expressed on T cells for homing to secondary lymphoid organs; necessary for central memory T cells to elicit recall responses upon antigen encounter |

| | |
|---|---|
| TNFRSF9/4-1BB | Also known as TNFR superfamily member 9 (TNFRSF9) or CD137; binds to TNFR-associated factor 2 (TRAF2); expressed on T cells, mainly CD8 T cells; essential for cytototoxic activity |
| ICOS/CD278 | Inducible T-cell costimulator; expressed on activated T cells |
| BTLA/CD272 | B- and T-lymphocyte attenuator; expressed on activated T cells and B cells; binds TNFR superfamily member 14 (TNFRS14) |
| VLA1/ITGA1-ITGB1 | Very-late-Antigen 1; also known as alpha integrin 1 beta integrin 1; collagen receptor expressed on memory CD4 T cells; also involved in T-cell penetration into the skin and lung, particularly in inflammation |
| CD27 | Interacts with CD70; expressed on activated T cells; has immune checkpoint properties |
| CD69 | Expressed on T cells and is involved in T-cell differentiation; also associated with retention of T cells in lymphoid organs as well as tissue residency |
| CD70 | Ligand for CD27; expressed on activated T cells |
| LAMP1/CD107a | Lysosome-associated membrane protein 1; mainly expressed on lysosomal membranes and plays a role in cytotoxicity of T and NK cells; can also be detected on the cell surface - serves as a marker of degranulation |
| CD16/FCGR3A | Although not expressed on the surface of T cells, CD16 (also known a Fcγ receptor III) expressed on the surface of tumour cells or other such target cells is needed for eliciting antibody-dependent cytotoxicity (ADCC) which involves NK cells and CD8 T cells; important for tumour control |
| ***Cytokines/chemokines*** | |
| IL1A/haematopoeitin 1 | IL-1α; produced by myeloid cells upon activation; involved in TNF-α synthesis |
| IL1B | IL-1β; produced by myeloid cells following inflammasome activation |
| IL2 | Produced by T cells as an essential growth factor |
| IL3 | Produced by T cells, mast cells and eosinophils; can activate APC functions and induce inflammation |
| IL4 | Produced by T cells; mainly involved in orchestration of Th2 responses and antibody production by B cells/plasma cells |
| IL5 | Anti-inflammatory cytokine produced by Th2 cells and mast cells; also involved in antibody production by B cells |
| IL6 | Pleiotropic cytokine released by activated myeloid cells; involved in pro-inflammatory activity by T cells; can also induce anti-inflammatory activity by Th2 |
| IL7 | Important CD4-centric gamma-chain cytokine required for their growth and maintenance of memory pools; produced by stromal cells, epithelial cells, hepatocytes, keratinocytes |
| IL8/CXCL8 | Important for neutrophil chemoattraction; produced by activated myeloid cells |
| IL9 | Produced by Th9 cells and may have a role in anti-tumour responses |

| | |
|---|---|
| IL10 | Generally anti-inflammatory in nature, IL-10 also has some inflammatory properties as seen in chronic diseases; produced by myeloid cells (mainly monocytes but also macrophages and dendritic cells, DCs) |
| IL11 | Produced mainly by stromal cells; important for megakaryocyte maturation |
| IL12 | A cardinal pro-inflammatory cytokine produced by activated macrophages and DCs; essential for production of IFN-gamma by Th1 cells, jointly with IL-18 |
| IL13 | Involved in the production of IgE antibodies and is secreted by several cells types; including Th2 cells, mast cells and NK T cells; also has a role in tumour progression |
| IL14 | Also known as alpha-taxilin; produced mainly by T cells and some malignant B cells; involved in expansion of B-cell subsets but inhibits antibody production |
| IL15 | Important gamma-chain cytokine involved in generation and maintenance of memory CD8 T cells; involved in promoting anti-tumour immune responses by memory T cells and NK cells |
| IL16 | Produced by various cell types, including T cells; potent chemoattractant for activated T cells |
| IL17 | Pro-inflammatory cytokine produced by Th17 cells and some gamma-delta (γδ) subsets; role in cancer remains controversial and the same for chronic infections |
| IL18 | Pro-inflammatory cytokine produced by M1 macrophages and DCs upon inflammasome activation; essential for inducing IFN-gamma production by Th1 cells jointly with IL-12 activity |
| IL19 | Produced by monocytes; may a have a role in orchestrating inflammation and apoptosis |
| IL20 | Produced by activated keratinocytes and monocytes; has a role in pro-inflammatory immune responses; belong to the IL-10 family of molecules |
| IL21 | Important gamma-chain cytokine involved in maintenance of immunological memory in pools of high-affinity CD8 T cells; essential for antiviral immune responses and downregulation of regulatory T-cell (Treg) responses |
| IL22 | Generally pro-inflammatory in nature; produced by innate lymphoid cells and Th22 cells; may have a role in promoting anti-tumour immune responses |
| IL23 | Comprises IL-12β and IL-23α subunits; involved in pro-inflammatory activity; produced by activated myeloid cells |
| IL24 | Pro-inflammatory cytokine produced by activated myeloid cells and Th2 cells; can induce cell death in responsive cells |
| IL25 | Also known as IL-17E and can induce NF-κB activation; produced by Th2 cells and mast cells during inflammation |
| IL26 | Produced by herpesvirus-infected T cells; induces expression of IL-10 and IL-8; structurally similar to IL-10 |

| | |
|---|---|
| IL27 | Comprises Epstein-Barr virus-induced gene 3 (EBI3 or IL-27β) and IL-27p28; produced by APCs; upregulates IL-10; may have a role in differentiation of T cells; previously known as IL-30 |
| IL28 | Two isoforms exists - IL-28A and IL-28B; type III interferon; very similar to IL-29 in structure; important in antiviral immune defence; produced by myeloid cells |
| IL29 | Very similar to IL-28 in structure; type III interferon; important in antiviral immune defence; produced by myeloid cells |
| IL31 | Has a pro-inflammatory activity profile but is produced by Th2 cells as well as other cell types |
| IL32 | Generally pro-inflammatory in nature; is involved in vitamin D3 and IFN-gamma signalling axis; produced by DCs and macrophages; may have a role in augmenting IL-15 activity and memory CD8 T-cell maintenance |
| IL33 | Produced by a myriad of cell types; including mast cells, activated myeloid cells, APCs; promotes IL-4 production by Th2 |
| IL34 | Produced largely by splenocytes; involved in promoting monocyte survival |
| IL35 | Produced by Tregs; comprises IL-12Rα and IL-27β; has immune-suppressive properties |
| IL36 | Three main agonistic forms (alpha, beta and gamma) and one antagonistic form (Rα) Produced by epithelial cells; can induce NF-κB and MAPK activation, and T-cell activtion as well as subsequent IL-2 production |
| IL37 | Belongs to IL-1 family; inhibits innate immune activation; produced by macrophages and epithelial cells |
| IL38 | Belongs to IL-1 family; produced by myeloid cells and a large variety of parenchymal cell types; inhibits IL-17 and IL-22 production by T cells |
| IL39 | Comprises IL-23p19 and EBI3; produced by activated B cells; has an effect on neutrophil expansion and activation |
| IL40 | Expressed by activated human B cells; may have an autocrine effect on B-cell expansion |
| IFNA1 | Interferon alpha (IFN-α); produced by activated myeloid cells; involved in initiation of inflammatory activity by innate immune cells and antiviral responses; might also play a role in anti-cancer responses |
| IFNA2 | Interferon alpha 2; important for antiviral defence; might play a role in STING pathway and anti-cancer responses |
| IFNA17 | Interferon alpha 17; important for antiviral defence; might play a role in STING pathway and anti-cancer responses |
| IFNB1 | Interferon beta (IFN-β); produced by fibroblasts; major role in amplifying innate immune responses and antiviral defence |
| IFNG | Interferon gamma (IFN-γ); Major Th1 cytokine also produced by other T cells with inflammatory properties as well as NK cells; important against viruses, tumours and intracellular bacteria |

| | |
|---|---|
| TNFA | Tumour necrosis factor alpha; crucical anti-tumour defence molecule, also against intracellular bacteria and viruses; produced by activated myeloid cells and T cells; induces apoptosis of target cells |
| CXCL1/SCYB1 | Produced by activated myeloid cells, neutrophils, epithelial cells and importanty melanoma cells; impacts on various physiological processes including angiogenesis and wound healing |
| CXCL2/SCYB2 | Also known as macrophage inhibitory protein 2 alpha (MIP2α); produced by monocytes and macrophages; chemoattractant for neutrophils and haematopoeitic stem cells |
| CXCL3/SCYB3 | Also known as macrophage inhibitory protein 2 betaa (MIP2β); produced by myeloid cells and epithelial cells; involved in monocyte migration and adhesion; also functions as a neutrophil chemoattractant |
| CXCL4/SCYB4 | Released by platelets; promotes blood coagulation; also involved in inflammation and wound healing |
| CXCL5/SCYB5 | Produced by epithelial cells; has inflammatory activity; expressed in response to IL-1β and TNF-α exposure |
| CXCL6/SCYB6 | Also known as granulocyte chemotactic protein 2 (GCP2); produced by endothelial cells; chemoattractant for neutrophils |
| CXCL7/SCYB7 | Produced by platelets; involved in mitogenesis, glucose metabolism, extracellular matrix synthesis; also neutrophil trafficking |
| CXCL8/SCYB8 | Also known as IL-8; produced by macrophages and epithelial cells; involved in neutrophil chemoattraction |
| CXCL9/SCYB9 | Produced by activated neutrophils and mast cells; T-cell chemoattractant; involved in amplification of Th1 responses in tissue, also CD8 T cell responses |
| CXCL10/SCYB10 | Also known as IFN-gamma-induced protein 10 kDa (IP-10); produced by activated marophages, monocytes and DCs; also by mast cells and neutrophils; strong T-cell chemoattractant but also chemotactic for monocytes, macrophages and DCs; involved in amplification of Th1 responses in tissue, also CD8 T cell responses |
| CXCL11/SCYB11 | Produced by mast cells; T-cell chemoattractant; involved in amplification of Th1 responses in tissue, also CD8 T cell responses |
| CXCL12/SCYB12 | Produced by CXCL12-abundant reticular cells and epithelial cells; functions as a B-cell chemoattractant; also involved in central memory T-cell trafficking |
| CXCL13/SCYB13 | Produced by ollicular DCs and mesencymal stromal cells; important function in the B-cell zone and interaction with T cells in lymph nodes |
| CXCL14/SCYB14 | Produced by fibroblasts; an important DC and monocyte chemoattractant |
| CXCL16/SCYB16 | Produced by DC in response to viral infections; involved in migration and survival of NKT cells as well as innate lymphoid cells |
| CXCL17/VCC1 | Constitutively expressed by lung cells; DC and monocyte chemoattractant |

| | |
|---|---|
| XCL1/SCYC1 | A type of lymphotactin; found in various tissue including spleen, intestines and peripheral blood; CD8 T cells in blood, NK cells and DCs also produce this chemokine; chemoattractant for T cells; macrophages and fibroblasts |
| XCL2/SCYC2 | A type of lymphotactin; produced mainly by T cells; chemoattractant for T cells; macrophages and fibroblasts |
| CX3CL1/SCYD1 | Also known as fractalkine; produced by activated DCs and macrophages; soluble and cell-bound versions exist; acts as a chemoattractant of T cells and monocytes |
| CCL1/SCYA1 | Produced by activated T cells; chemoattractant for monocytes, DCs, NK cells and immature B cells; also involved in Th2 cell and Treg trafficking |
| CCL2/SCYA2 | Also known as monocyte chemoattractant protein 1 (MCP-1); produced by activated myeloid cells; chemoattractant for monocytes, DCs and T cells; involved in amplifying the inflammatory response |
| CCL3/SCYA3 | Also known as macrophage inflammatory protein 1-alpha (MIP-1α); produced by activated macrophages; is chemotactic for neutrophils |
| CCL4/SCYA4 | Also known as macrophage inflammatory protein 1-beta (MIP-1β); produced by monocytes, B Cells, T Cells, fibroblasts, endothelial as well as epithelial cells.; is chemotactic for monocytes, NK cells |
| CCL5/SCYA5 | Also known as RANTES; produced by Th1 cells and CD8 T cells; can activated NK cells and myeloid cells (also APCs); has suppressive effects on HIV-infected cells |
| CCL7/SCYA7 | Produced by macrophages and some tumour cell lines as well as stromal cells; chemoattractant for monocytes and regulation of T-cell function |
| CCL8/SCYA8 | Also known as monocyte chemoattractant protein 2 (MCP-2); produced by fibroblasts and endothelial cells; chemotactic for various immune cell types including T cells, NK cells, mast cells, monocytes; also involved in T-cell homing to skin |
| CCL9/SCYA9 | Previously known as CCL10; now also known as macrophage inflammatory protein-1 gamma (MIP-1γ); produced by macrophages and other myeloid cell types; also by follicle-associated epithelial cells |
| CCL11/SCYA11 | Also known as eotaxin; produced by stromal cells and activated myeloid cells; chemotactic for eosinophils |
| CCL13/SCYA13 | Also known as monocyte chemotactic protein 4 (MCP-4); produced by airway epithelial cells; chemotactic for eosinophils; T cells; basophils; monocytes |
| CCL14/SCYA14 | Produced by various cells types including splenocytes, myocyes and hepatocytes; activates monocytes |
| CCL15/SCYA15 | Also known as leukotactin-1; expressed by cells in the liver and gut; function is not very clear |
| CCL16/SCYA16 | Also known as monotactin-1; produced by cells in the liver, thymus and spleen; chemotactic for lymphocytes and monocytes |

| | |
|---|---|
| CCL17/SCYA17 | Also known as thymus and activation regulated chemokine (TARC); produced in thymic cells; is chemotactic for T cells; |
| CCL18/SCYA18 | Produced mainly by monocytes, DCs and macrophages; chemotactic for T cells and immature B cells; also DCs |
| CCL19/SCYA19 | Produced by activated macrophages and DCs; involved in thymic T-cell trafficking and lymphocyte migration to secondary lymphoid organs; also attracts CCR7+ central memory T cells |
| CCL20/SCYA20 | Also known as macrophage inflammatory protein 3 (MIP-3A); chemotactic for lymphocytes; also DCs to a lesser extent; produced by various cells types including APCs |
| CCL21/SCYA20 | Produced by stromal cells and fibroblastic reticular cells; cell-bound form of CCL21 expressed by activated DCs; involved in homing of T cells and DCs to lymph nodes |
| CCL22/SCYA22 | Produced by DCs and macrophages; important for Th2 and Treg migration into lymph nodes during inflammation |
| CCL23/SCYA23 | Also known as myeloid progenitor inhibitory factor 1 (MPIF-1); expressed mainly by cells in the lung and liver; chemotactic for resting T cells and monocytes; lesser extent for neutrophils |
| CCL24/SCYA24 | Also known as myeloid progenitor inhibitory factor 2 (MPIF-2); chemotactic for resting T cells and to lesser extent for neutrophils |
| CCL25/SCYA25 | Also known as thymus-expressed chemokine (TECK); produced by epithelial cells; involved in T-cell development; chemotactic for macrophages, DCs and thymocytes |
| CCL26/SCYA26 | Also known as eotaxin-3; expressed by several cell types including heart and liver cells; chemotactic for neutrophils and basophils |
| CCL27/SCYA27 | Also known as cutaneous T-cell-attracting chemokine (CTACK); expressed in several tissue types including thymus, placenta and skin; involved in T-cell trafficking to the skin |
| CCL28/SCYA28 | Also known as mucosae-associated epithelial chemokine (MEC); produced by several tissue types including gut, lung and breast involved in T-cell and IgA+ plasma cell trafficking/homing to mucosal tissues i.e. pulmonary, mammary glands; intestines |
| CSF2 | Also known as granulocyte-macrophage colony-stimulating factor (GM-CSF); important for differentiation of macrophages from monocytes; T cells also respond to this cytokine and in fact produce them; also DCs, mast cells, fibroblasts and NK cells |
| LTA/TNFB | Lymphotoxin alpha (LTα); previously known as TNF-β; produced by T cells; enhances cell adhesion to endothelia, thus cell migtation and trafficking; also involved in apoptosis induction, similar to TNF-α |
| LTB/TNFC | Lymphotoxin beta (LTβ); previously known as TNF-C; type II membrane-bound protein; can form a heterodimer with LTα; involved in apoptosis induction |
| ***Cytokine/chemokine receptors*** | |
| IFNAR1 | Interferon alpha receptor 1; binds IFN-α/β; expressed on T cells |

| | |
|---|---|
| IFNAR2 | Interferon alpha receptor 2; binds IFN-α/β; expressed on T cells |
| IFNGR1 | Interferon gamma receptor 1; mandatory for IFN-gamma signalling in cells |
| IFNGR2 | Interferon gamma receptor 2; mandatory for IFN-gamma signalling in cells |
| TNFRSF1A | Tumor necrosis factor receptor superfamily member 1A; binds to and is essential for TNF-alpha signalling in cells |
| TNFRSF1B | Tumor necrosis factor receptor superfamily member 1B; binds to and is essential for TNF-alpha signalling in cells |
| IL1R1/CD121A | Interleukin 1 receptor; important for IL-1β-mediated pro-inflammatory activity produced by inflammasome activation; expresse on T cells |
| IL2RA/CD25 | IL-2 receptor alpha subunit; binds to IL-2, the most important T-cell growth factor produced by T cells themselves. |
| IL2RB | IL-2 receptor beta subunit; forms a trimer with CD25 and CD132 to constitute the IL-2R complex for binding IL-2 |
| IL2RG/CD132 | IL-2 receptor gamma subunit; also known as common gamma chain, essential for gamma-chain cytokine signalling |
| IL3RA/CD123 | IL-3 receptor alpha; forms a dimer with CD131 (common beta chain) to bind IL-3; expressed on pluripotent progenitor cells |
| IL4R/CD124 | IL-4 receptor; necessary for binding to IL-4 and downstream signalling; also binds to IL-13 |
| IL5RA/CD125 | IL-5 receptor alpha subunit; forms a dimer with CSF2RB to form IL-5R; binds IL-5; expressed on eosinophils and some basophils |
| IL5RB/CSF2RB | IL-5 receptor beta subunit; forms a dimer with CSF2RB to form IL-5R; binds IL-5, IL-3 and GM-CSF; expressed on leukocytes |
| IL6R/CD126 | IL-6 receptor; forms a dimer with gp130 to bind IL-6 and is necessary for signalling in cells |
| IL6ST/gp130 | IL-6 signal transducer; also known as glycoprotein 130 (gp130); forms a dimer with CD126/IL6R for IL-6 signalling in cells |
| IL7R/CD127 | IL-7 receptor alpha subunit; essential for IL-7 signalling; forms a dimer with CD132; also a marker for memory CD4 T cells |
| IL8RA/CXCR1 | IL-8 receptor alpha subunit; important for IL-8 signalling, expressed on neutrophils |
| IL9R/CD129 | IL-9 receptor; forms a complex with CD132 to bind IL-9; is found on a variety of cell types |
| IL10RA/CD210a | IL-10 receptor alpha subunit; expressed on haematopoeitic cells; important for IL-10 signalling |
| IL10RB/CDw210b | IL-10 receptor beta subunit; ubiquitously expressed; important for IL-10 signalling; also forms a dimer with IL-20Rα to bind IL-26 |
| IL11RA/CRSDA | IL-11 receptor alpha subunit; forms a dimer with gp130 to bind IL-11; expressed on haematopoeitic cells and signalling is essential for early development in the bone marrow |

| | |
|---|---|
| IL12RB1/CD212 | IL-12 receptor beta subunit 1; forms a dimer with IL-12Rβ2; necessary for binding to IL-12; expressed on T cells and needed for inducing IFN-gamma production jointly with IL-18 |
| IL12RB2 | IL-12 receptor beta subunit 2; necessary for binding to IL-12; expressed on T cells and needed for inducing IFN-gamma production jointly with IL-18; may also form a dimer with gp130 to bind IL-35 but the downstream signalling suppresses inflammation |
| IL13RA1/CD213A1 | IL-13 receptor alpha subunit 1; forms a dimer with IL-4Rα to bind IL-13; also binds IL-4; expressed on B cells |
| IL13RA2/CD213A2 | IL-13 receptor alpha subunit 2; binds IL-13 with higher affinity than IL-13Rα1; expressed on some tumour cell types i.e. glioma |
| IL14R | Binds to IL-14 (also known as alpha-taxilin) produced by T cells; expressed on B cells and required for activation, cytokine and antibody production |
| IL15RA/CD215 | IL-15 receptor alpha subunit; forms a complex with IL-2Rβ and CD132 to bind to IL-15 for signalling; expressed on T cells |
| CD4 as IL-16R | The CD4 co-receptor acts as a receptor for IL-16 and is considered important for CD4 T-cell chemoattraction |
| IL17RA/CD217 | IL-17 receptor alpha subunit; involved in IL-17 binding and downstream signalling; usually part of a heteromeric structure consisting of several other members i.e. IL-17Rβ, IL-17Rc; expressed on many cell types and also on CD8 T cells |
| IL17RB/CRL4 | IL-17 receptor beta subunit; involved in heterodimersation with IL-17R members to bind IL-17; also IL-25 (IL-17E) |
| IL18RA/CD218a | IL-18 receptor alpha; important for pro-inflammatory activity and IFN-γ production jointly with IL-12; produced by inflammasome activation; may also bind IL-37 with IL-1 receptor 8 |
| IL20RA/CRF2-8 | IL-20 receptor alpha subunit; forms a dimer with IL-20Rβ to bind IL-20; expressed on keratinocytes and epithelial cells; also required for IL-19 and IL-24 signalling; dimerises with IL-10Rβ to bind IL-26 |
| IL20RB/DIRS1 | IL-20 receptor beta subunit; forms a dimer with IL-20Rα to bind IL-20; expressed on keratinocytes and epithelial cells; also required for IL-19 and IL-24 signalling |
| IL21R/CD360 | IL-21 receptor; forms a dimer with the common gamma chain (CD132) and binds to IL-21 for downstream signalling; expressed on T cells, particularly high-affinity memory subsets |
| IL22RA1/CRF2-9 | IL-22 receptor alpha subunit 1; forms a dimer with IL-10Rβ2 for binding IL-22 and downstream signalling; important for pro-inflammatory activity; expressed on parenchymal cells but not on immune cells; also involved in IL-24 signalling |
| IL23RA | IL-23 receptor alpha; forms a dimer with IL-12Rβ1 to bind IL-23; expressed on immune cells, including myeloid cells and Th17 cells; also binds IL-39 |

| | |
|---|---|
| IL27RA/CRL1 | IL-27 receptor alpha subunit; forms a dimer with gp130 to bind IL-27 (also known as IL-30); expressed on T cells |
| IL28RA/IFNLR1 | IL-28 receptor alpha; also known as IFN lambda receptor 1; forms a dimer with IL-10Rβ2 for binding IL-28 and IL-29; expressed on T cells and plays a role in augmenting antigen-specific CD8 T-cell responses |
| IL31RA/CRL3 | IL-31 receptor alpha subunit; similar to gp130, and is expressed on monocytes; important for IL-31 binding and signalling |
| IL32R | Probably an intracellular receptor that bind IL-32 (alpha, beta, gamma) |
| IL33R/IL1RL1 | IL-1 receptor-like 1 (IL-1RL1); binds IL-33; expressed on T cells and innate lymphoid cells; signalling is important for Th2 development |
| IL34R/PTP-ζ | Receptor-type protein-tyrosine phosphatase zeta; binds IL-34; expressed on neuronal progenitors and glial cells |
| IL36R/IL1RL2 | IL-1 receptor-like 2 (IL-1RL2); forms a dimer with IL-1 receptor-like 2 (IL-1RL2) to bind IL-36; expressed on T cells; also binds IL-38 |
| IL40R | Binds IL-40; yet to be described |
| TRAILR1/TNFRSF10A | TNF receptor superfamily member 10a; forms a dimer with TRAILR2 to bind TRAIL for induction of apoptosis; expressed on a variety of cell types |
| TRAILR2/TNFRSF10B | TNF receptor superfamily member 10b; forms a dimer with TRAILR1 to bind TRAIL for induction of apoptosis; expressed on a variety of cell types |
| TRAILR3/TNFRSF10C | TNF receptor superfamily member 10c; forms a dimer with TRAILR4 to bind TRAIL for subverting apoptosis induction; expressed on a variety on cell types |
| TRAILR4/TNFRSF10D | TNF receptor superfamily member 1d; forms a dimer with TRAILR3 to bind TRAIL for subverting apoptosis induction; expressed on a variety on cell types |
| OPG/TNFRSF11B | Osteoprogeterin; also known as TNF receptor superfamily ember 11b; binds TRAIL; expressed on innate immune cells |
| CCR1 | Chemokine receptor CCR1; binds CXCL3, 4, 6, 8, 9, 10, 14, 15 ,16, 23 |
| CCR2 | Chemokine receptor CCR2; binds CCL2, 7, 11, 13, 16 |
| CCR3 | Chemokine receptor CCR3; binds CCL11, 13, 15, 24, 26, 28 |
| CCR4/CD194 | Chemokine receptor CCR4; binds CCL2 (MCP-1), CCL4 (MIP-1), CCL5 (RANTES), CCL17 (TARC), CCL22 (Macrophage-derived chemokine); involved in monocyte chemoattraction and activation; also in identification of Th2 cells; also involved in T-cell penetration into cardiac, lung and skin tissue |
| CCR5/CD195 | Chemokine receptor CCR5; also important for HIV binding and entry into CD4 T cells; binds CCL4, 5, 8, 11, 13, 16, |

| | |
|---|---|
| CCR6/CD196 | Chemokine receptor CCR6; binds MIP-3α; also important for identifcation of Th17 cells and Th1* cells (with CCR4); also involved in T-cell penetration into the lung |
| CCR7/CD197 | Chemokine receptor CCR7; binds CXCL20 and CXCL21; also a marker for central memory T cells |
| CCR8/CDw198 | Chemokine receptor CCR8; binds CCL1, 16 |
| CCR9/CDw199 | Chemokine receptor CCR9; binds CCL25; also involved in T-cell penetration into the gut and liver |
| CCR10/GPR2 | Chemokine receptor CCR10; binds CCL28, 28 |
| CXCR1/CD181 | Chemokine receptor CXCR1; binds CXCL6, 8 |
| CXCR2/IL8RB | Chemokine receptor CXCR2; also known as IL.8 receptor beta; binds CXCL1, 2, 3, 5, 6, 8 |
| CXCR3A/GPR9 | Chemokine receptor CXCR3; binds CXCL9, 10 and 11; CXCR3B |
| CXCR3B | Chemokine receptor CXCR3A isoform CXCR3B; binds CXCL4,9,10,11. Also involved in T-cell penetration into lung and cardiac tissue, and identification of Th1 cells |
| CXCR4/CD184 | Chemokine receptor CXCR4; binds SDF-1/CXCL12; important for T-cell penetration into the lung |
| CXCR5/CD185 | Chemokine receptor CXCR5; binds CXCL13; involved in T-cell migration to B-cell zones in lymph nodes |
| CXCR6/CD186 | Chemokine receptor CXCR6; binds CXCL16; involved in T-cell penetration into the liver |
| CXCR7/ACKR3 | Chemokine receptor CXCR7; also known as atypical chemokine receptor 3; binds CXCL11, 12 |
| TGFBR1/ALK5 | Transforming growth factor beta receptor 1; also known as anaplastic lymphoma kinase 5 (ALK5); binds with affinity to TGF-β1 |
| TGFBR2 | Transforming growth factor beta receptor 2; binds with affinity to TGF-β1 |
| CD117/KIT | Mast/stem cell growth factor receptor (SCFR); also known as c-Kit; expressed on haematopoietic precursors as well as some antigen-specific precursor CD8 T cells; important growth factor |
| LTBR/CD18 | Also known as TNFR superfamily member 3 (TNFRSF3); binds lymphotoxin; expressed on various cell types including parenchymal and stromal cells |
| VLA4/ITGA4-ITGB1 | Very-late-antigen 4, comprising alpha integrin 4 and beta integrin 1; binds VCAM-1, SDF-1 and fibronectin; involved in T-cell traffciking to the brain |
| XCR1 | Receptor for lymphotactins XCL1 and XCL2; expressed on T cells, macrophages, fibroblasts |
| ***Toll-like receptors*** | |
| TLR1/CD281 | Toll-like receptor 1; forms a dimer with TLR2 to recognise peptidoglycan; expressed on the cell surface |
| TLR2/CD282 | Toll-like receptor 2; forms a dimer with TLR1 to recognise peptidoglycan; also recognises a myriad of bacterial cell components; expressed on the cell surface; regulates CYP1A1 activity in the intestine |
| TLR3/CD283 | Toll-like receptor 3; recognises dsRNA of viral origin; expressed intracellularly |
| TLR4/CD284 | Toll-like receptor 4; recognises bacterial lipopolysaccharide; important for NF-κB activation and subsequent TNF-α production by APCs; expressed on the cell surface |

| | |
|---|---|
| TLR5/MELIOS | Toll-like receptor 5; recognises bacterial flagellin; also implicated in NF-κB activation and subsequent TNF-α production by APCs |
| TLR6/CD286 | Toll-like receptor 6; synergises with TLR2 to recognise bacterial lipoproteins; expressed on the cell surface |
| TLR7 | Toll-like receptor 7; recognises endosome-encapsulated ssRNA; generally expressed intracellularly |
| TLR8/CD288 | Toll-like receptor 8; endosomal molecule which recognises ssRNA, akin to TLR7; expressed intracellularly |
| TLR9/CD289 | Toll-like receptor 9; recognises bacterial and viral DNA; expressed intracellularly |
| TLR10/CD290 | Toll-like receptor 10; shown to be expressed on B cells and neutrophils; expressed intracellularly in epithelial cells; has anti-inflammatory and inflammatory downstream effects; likely to activated by bacterial ligand i.e. *Listeria monocytogenes* components |
| ***Immune-related intracellular molecules*** | |
| ZAP-70 | Zeta-chain-associated protein kinase 70; expressed intracellularly in T cells; involved in TCR signalling and T-cell activation |
| MyD88 | Myeloid differentiation primary response 88; adapter molecule indispensable for TLR2/4-mediated anti-microbial protection |
| NF-κB | Nuclear factor kappa-light-chain-enhancer of activated B cells; essential for TLR4-mediated primary immune responses |
| IKBKB | Inhibitor Of Nuclear Factor Kappa B Kinase Subunit Beta; phosphorylates the inhibitor in the inhibitor/NF-κB complex, leading to dissociation of the inhibitor and NF-κB activation |
| STING/TMEM173 | Stimulator of interferon genes; found in many cell types; important for intracellular pro-inflammatory activity |
| TBK1 | TANK-binding kinase 1; necessary for phosphorylation of STING to activate it |
| CGAS | Cyclic GMP-AMP synthase; generates cyclic GMP-AMP leads to STING activation in cells |
| IDO1 | Indoleamine 2,3-dioxygenase 1; heme-containing enzyme important in converting typtophan to kynurenines; upregulated by IFN-gamma signalling and has immuno-suppressive function |
| IDO2 | Indoleamine 2,3-dioxygenase 2; heme-containing enzyme important in converting typtophan to kynurenines; upregulated by IFN-gamma signalling and has immuno-suppressive function |
| JAK1 | Janus kinase 1; involved in IFN and other pro-inflammatory cytokine signalling pathways |
| JAK2 | Janus kinase 2; involved in IFN and other pro-inflammatory cytokine signalling pathways |
| STAT1 | Signal transducer and activator of transcription 1; involved in IFN-mediated inflammatory cell signalling in conjuction with JAK1/2 |
| STAT2 | Signal transducer and activator of transcription 2; involved in IFN-γ signalling pathway |

| | |
|---|---|
| STAT3 | Signal transducer and activator of transcription 3; various cytokine-mediated cell signalling pathways; also important for Th17 activation |
| STAT4 | Signal transducer and activator of transcription 4; important for Th1 development |
| STAT5 | Signal transducer and activator of transcription 5; important for IL-2 and IL-7-mediated cell proliferation (T cells); also activated downstream of CSF2/GM-CSF signalling |
| STAT6 | Signal transducer and activator of transcription 6; involved in Th2 responses and induction of an anti-apoptosis state in cells |
| MAPK1 | Mitogen-activated protein kinase 1; an extracellular signal-regulated kinase (ERK); involved in various cellular processes; important in pro-inflammatory signalling i.e. IL-6, TNF-alpha, IL-18, type 1 IFNs |
| IRF1 | Interferon regulatory factor 1; involved in amplification of JAK/STAT pathway after IFN-gamma signalling initiates; CD8 T cells responses and Th1 responses |
| IRF2 | Interferon regulatory factor 2; activation inhibits IFN-alpha/beta production |
| IRF3 | Interferon regulatory factor 3; activated by TLR4 signalling and thus may play arole in antibacterial defence; pro-apoptotic properties |
| IRF4 | Interferon regulatory factor 4; has a role in MHC-I regulation and thus, CD8 T-cell responses |
| IRF5 | Interferon regulatory factor 5; involved in induction of inflammation in response to viruses; apoptosis of infected cells; amplifiction of primary (innate) responses |
| IRF6 | Interferon regulatory factor 6; has development roles in skin tissue, and potentially tumour-suppressive properties |
| IRF7 | Interferon regulatory factor 7; involved in type I interferon production and antiviral defence; constitutively expressed in lymphoid tissue |
| IRF8 | Interferon regulatory factor 8; involved in myeloid-cell differentiation, at the commitment step where common lymphoid progenitors (CMP) differentiate into monocyte precursors |
| IRF9 | Interferon regulatory factor 9; interacts with STAT1/2; promotes the cellular toxicity of IFN-alpha potentially through TRAIL activity |
| IRF10 | Interferon regulatory factor 10; downstream of IFN-gamma signalling and involved in MHC-I regulation; not associated with cytotoxicity |
| NLRP3 | NACHT, LRR and PYD domains-containing protein 3 (NALP3); essential for inflammasome activation |
| NLRP4 | NACHT, LRR and PYD domains-containing protein 3 (NALP4); also involved in inflammasome activation |
| MDA-5 | Melanoma Differentiation-Associated protein 5; recognises intracellular dsRNA |
| RIG-I | Retinoic acid-inducible gene I; dsRNA helicase enzyme with a prominent role in protection against viral infections; may play a role in augmenting clinical responses to cancer therapies |
| RAG-1/2 | Recombination-activating genes 1/2; indispensable for rearrangement of TCR and BCR |

| | |
|---|---|
| Caspases | Intracellular enzymes with a myriad of functions, including processing of pro-inflamatory cytokines such as IL-1β to initiate and/or amplify immune responses |
| Granzyme B | Important cytotoxic effector molecule produced by CD8 T cells and NK cells; involved in lysing target cells via apoptosis |
| Perforin | Important effector molecules produced by cytotoxic T cells and NK cells; causes perforations in target cells to allow granzyme penetration to trigger apoptosis |
| Granulysin | Lytic molecule produced by cytotoxic lymphocytes including CD8 T cells and γδ T cells |
| TBX21 | Also known as T-bet; crucial transcription factor for Th1 maturation and production of IFN-gamma; may also negativelyt regulate Th22 development |
| GATA3 | GATA-3 transcription factor necessary for Th2 polarisation and associated cytokine production by T cells i.e. IL-4, IL-5, IL-13 |
| RORC | Encodes RORγt, transcription factor necessary for Th17 and Th22 development |
| AHR | Aryl hydrocarbon receptor; transcription factor which recognises dioxin-like compound intracellularly and in response induces pro-inflammatory responses; important in antibacterial defence |
| ***DNA repair genes (taken directly from Wood et al., 2001. Science. 291: 1284-89)*** | |
| **Base excision repair (BER)** | |
| **DNA glycosylases: major altered base released** | |
| UNG | U |
| SMUG1 | U |
| MBD4 | U or T opposite G at CpG sequences |
| TDG | U, T or ethenoC opposite G |
| OGG1 | 8-oxoG opposite C |
| MYH | A opposite 8-oxoG |
| NTH1 | Ring-saturated or fragmented pyrimidines |
| MPG | 3-meA, ethenoA, hypoxanthine |
| **Other BER factors** | |
| APE1 (HAP1, APEX, REF1) | AP endonuclease |
| APE2 (APEXL2) | AP endonuclease |
| LIG3 | Main ligation function |
| XRCC1 | Main ligation function |
| Poly(ADP-ribose) polymerase (PARP) enzymes | |
| ADPRT | Protects strand interruptions |
| ADPRTL2 | PARP-like enzyme |

| | |
|---|---|
| ADPRTL3 | PARP-like enzyme |
| **Direct reversal of damage** | |
| MGMT | O6-meG alkyltransferase |
| **Mismatch excision repair (MMR)** | |
| MSH2 | Mismatch and loop recognition |
| MSH3 | Mismatch and loop recognition |
| MSH6 | Mismatch recognition |
| MSH4 | MutS homolog specialized for meiosis |
| MSH5 | MutS homolog specialized for meiosis |
| PMS1 | Mitochondrial MutL homolog |
| MLH1 | MutL homolog |
| PMS2 | MutL homolog |
| MLH3 | MutL homolog of unknown function |
| PMS2L3 | MutL homolog of unknown function |
| PMS2L4 | MutL homolog of unknown function |
| **Nucleotide excision repair (NER)** | |
| XPC | Binds damaged DNA as complex |
| RAD23B (HR23B) | Binds damaged DNA as complex |
| CETN2 | Binds damaged DNA as complex |
| RAD23A (HR23A) | Substitutes for HR23B |
| XPA | Binds damaged DNA in preincision complex |
| RPA1 | Binds DNA in preincision complex |
| RPA2 | Binds DNA in preincision complex |
| RPA3 | Binds DNA in preincision complex |
| TFIIH | Catalyzes unwinding in preincision complex |
| XPB (ERCC3) | 3' to 5' DNA helicase |
| XPD (ERCC2) | 5' to 3' DNA helicase |
| GTF2H1 | Core TFIIH subunit p62 |
| GTF2H2 | Core TFIIH subunit p44 |
| GTF2H3 | Core TFIIH subunit p34 |
| GTF2H4 | Core TFIIH subunit p52 |
| CDK7 | Kinase subunit of TFIIH |
| CCNH | Kinase subunit of TFIIH |
| MNAT1 | Kinase subunit of TFIIH |

| | |
|---|---|
| XPG (ERCC5) | 3' incision |
| ERCC1 | 5' incision subunit |
| XPF (ERCC4) | 5' incision subunit |
| LIG1 | DNA joining |
| **NER-related** | |
| CSA (CKN1) | Cockayne syndrome; needed for transcription-coupled NER |
| CSB (ERCC6) | Cockayne syndrome; needed for transcription-coupled NER |
| XAB2 (HCNP) | Cockayne syndrome; needed for transcription-coupled NER |
| DDB1 | Complex defective in XP group E |
| DDB2 | Mutated in XP group E |
| MMS19 | Transcription and NER |
| **Homologous recombination** | |
| RAD51 | Homologous pairing |
| RAD51L1 (RAD51B) | Rad51 homolog |
| RAD51C | Rad51 homolog |
| RAD51L3 (RAD51D) | Rad51 homolog |
| DMC1 | Rad51 homolog, meiosis |
| XRCC2 | DNA break and cross-link repair |
| XRCC3 | DNA break and cross-link repair |
| RAD52 | Accessory factor for recombination |
| RAD54L | Accessory factor for recombination |
| RAD54B | Accessory factor for recombination |
| BRCA1 | Accessory factor for transcription and recombination |
| BRCA2 | Cooperation with RAD51, essential function |
| RAD50 | ATPase in complex with MRE11A, NBS1 |
| MRE11A | 3' exonuclease |
| NBS1 | Mutated in Nijmegen breakage syndrome |
| **Nonhomologous end-joining** | |
| Ku70 (G22P1) | DNA end binding |
| Ku80 (XRCC5) | DNA end binding |
| PRKDC | DNA-dependent protein kinase catalytic subunit |
| LIG4 | Nonhomologous end-joining |
| XRCC4 | Nonhomologous end-joining |
| | Sanitization of nucleotide pools |

| | |
|---|---|
| MTH1 (NUDT1) | 8-oxoGTPase |
| DUT | dUTPase |
| **DNA polymerases (catalytic subunits)** | |
| POLB | BER in nuclear DNA |
| POLG | BER in mitochondrial DNA |
| POLD1 | NER and MMR |
| POLE1 | NER and MMR |
| PCNA | Sliding clamp for pol delta and pol epsilon |
| REV3L (POLZ) | DNA pol zeta catalytic subunit, essential function |
| REV7 (MAD2L2) | DNA pol zeta subunit |
| REV1 | dCMP transferase |
| POLH | XP variant |
| POLI (RAD30B) | Lesion bypass |
| POLQ | DNA cross-link repair |
| DINB1 (POLK) | Lesion bypass |
| POLL | Meiotic function |
| POLM | Presumed specialized lymphoid function |
| TRF4-1 | Sister-chromatid cohesion |
| TRF4-2 | Sister-chromatid cohesion |
| **Editing and processing nucleases** | |
| FEN1 (DNase IV) | 5' nuclease |
| TREX1 (DNase III) | 3' exonuclease |
| TREX2 | 3' exonuclease |
| EX01 (HEX1) | 5' exonuclease |
| SPO11 | endonuclease |
| **Rad6 pathway** | |
| UBE2A (RAD6A) | Ubiquitin-conjugating enzyme |
| UBE2B (RAD6B) | Ubiquitin-conjugating enzyme |
| RAD18 | Assists repair or replication of damaged DNA |
| UBE2VE (MMS2) | Ubiquitin-conjugating complex |
| UBE2N (UBC13, BTG1) | Ubiquitin-conjugating complex |
| **Genes defective in diseases associated with sensitivity to DNA damaging agents** | |
| BLM | Bloom syndrome helicase |
| WRN | Werner syndrome helicase/3'-exonuclease |

| | |
|---|---|
| RECQL4 | Rothmund-Thompson syndrome |
| ATM | Ataxia telangiectasia |
| **Fanconi anemia** | |
| FANCA | Involved in tolerance or repair of DNA cross-links |
| FANCB | Involved in tolerance or repair of DNA cross-links |
| FANCC | Involved in tolerance or repair of DNA cross-links |
| FANCD | Involved in tolerance or repair of DNA cross-links |
| FANCE | Involved in tolerance or repair of DNA cross-links |
| FANCF | Involved in tolerance or repair of DNA cross-links |
| FANCG (XRCC9) | Involved in tolerance or repair of DNA cross-links |
| **Other identified genes with a suspected DNA repair function** | |
| SNM1 (PS02) | DNA cross-link repair |
| SNM1B | Related to SNM1 |
| SNM1C | Related to SNM1 |
| RPA4 | Similar to RPA2 |
| ABH (ALKB) | Resistance to alkylation damage |
| PNKP | Converts some DNA breaks to ligatable ends |
| **Other conserved DNA damage response genes** | |
| ATR | ATM- and PI-3K–like essential kinase |
| RAD1 (S. pombe) homolog | PCNA-like DNA damage sensor |
| RAD9 (S. pombe) homolog | PCNA-like DNA damage sensor |
| HUS1 (S. pombe) homolog | PCNA-like DNA damage sensor |
| RAD17 (RAD24) | RFC-like DNA damage sensor |
| TP53BP1 | BRCT protein |
| CHEK1 | Effector kinase |
| CHK2 (Rad53) | Effector kinase |
